# Mathematical modelling of transcriptional heterogeneity identifies novel markers and subpopulations in complex tissues

**DOI:** 10.1038/srep18909

**Published:** 2016-01-07

**Authors:** Niya Wang, Eric P. Hoffman, Lulu Chen, Li Chen, Zhen Zhang, Chunyu Liu, Guoqiang Yu, David M. Herrington, Robert Clarke, Yue Wang

**Affiliations:** 1 Department of Electrical and Computer Engineering, Virginia Polytechnic Institute and State University, Arlington, VA 22203, USA; 2 Research Center for Genetic Medicine, Children’s National Medical Center, Washington, DC 20007, USA; 3 Pediatric Oncology Branch, National Institutes of Health, Gaithersburg, MD 20877, USA; 4 Department of Pathology, Johns Hopkins University, Baltimore, MD 21231, USA; 5 Department of Psychiatry, University of Illinois at Chicago, Chicago, Illinois 60607, USA; 6 Department of Medicine, Wake Forest University, Winston-Salem, NC 27157, USA; 7 Lombardi Comprehensive Cancer Center, Georgetown University, Washington, DC 20057, USA

## Abstract

Tissue heterogeneity is both a major confounding factor and an underexploited information source. While a handful of reports have demonstrated the potential of supervised computational methods to deconvolute tissue heterogeneity, these approaches require *a priori* information on the marker genes or composition of known subpopulations. To address the critical problem of the absence of validated marker genes for many (including novel) subpopulations, we describe convex analysis of mixtures (CAM), a fully unsupervised in silico method, for identifying subpopulation marker genes directly from the original mixed gene expressions in scatter space that can improve molecular analyses in many biological contexts. Validated with predesigned mixtures, CAM on the gene expression data from peripheral leukocytes, brain tissue, and yeast cell cycle, revealed novel marker genes that were otherwise undetectable using existing methods. Importantly, CAM requires no *a priori* information on the number, identity, or composition of the subpopulations present in mixed samples, and does not require the presence of pure subpopulations in sample space. This advantage is significant in that CAM can achieve all of its goals using only a small number of heterogeneous samples, and is more powerful to distinguish between phenotypically similar subpopulations.

Tissue heterogeneity, arising from multiple subpopulations within a sample, is both a major confounding factor in studying individual subpopulations and an underexploited information source for characterizing complex tissues^1^,^2^. Since the interactions among subpopulations are fundamental to both normal development and disease progression, molecular analysis of subpopulations in their native microenvironment provides the most biologically relevant picture of the *in vivo* state^3^,^4^. Complex tissues can be characterized by the identity, composition, and expression profile of possibly unknown subpopulations^5^, where subpopulations are often defined by marker genes (genes whose expressions are exclusively enriched in a particular subpopulation^6^,^7^, Fig. 1a). Current global profiling methods can neither identify differentially expressed genes among different subpopulations, nor distinguish among the contributions of different subpopulations to a globally measured gene expression profile^1^,^5^. Thus, it is generally impossible to tell whether expression change reflects a change in subpopulation composition, a change in subpopulation-specific expression, or both.

An experimental solution to mitigate tissue heterogeneity is to isolate subpopulations before molecular profiling by supervised cell sorting or tissue microdissection^1^,^8^. However, these methods are biased, costly, inapplicable to previously-assayed samples, and may alter the expression values^5^,^6^, restricted to cell-types that can be separated from the others. While some reports have demonstrated the potential of computational methods to resolve tissue heterogeneity, *a priori* information on the composition^2^,^5^,^9^ or signatures^6^,^10^,^11^,^12^ of the subpopulations believed to be present is almost exclusively required. Acquiring these prior information relies on experimental solutions and key limitations remain unsolved. Such supervised methods consequently have difficulty detecting subpopulations that are subtle, condition-specific (molecular signatures and cell function are changed but not cell appearance), or previously unknown^3^,^13^.

To address the critical problem of the absence of validated marker genes for many (including novel) subpopulations, we developed a fully unsupervised computational method (convex analysis of mixtures – CAM) that can identify these marker genes directly from the original mixed expressions in scatter space-a nontrivial task. CAM requires no prior information on the number, identity, or composition of the subpopulations present in mixed samples^12^, and does not require the presence of pure subpopulations in sample space^14^,^15^. Fundamental to the success of our approach is the newly-proven mathematical theorems, showing that the scatter simplex of mixed expressions is a rotated and compressed version of the scatter simplex of pure expressions, where the marker genes are located at each vertex (Fig. 1b). CAM works by geometrically identifying the vertices (and their resident genes) of the scatter simplex of globally measured expressions (Methods).

Tissue samples to be analyzed by CAM contain unknown numbers and varying proportions of distinct subpopulations. Expression of a given gene in a specific subpopulation is modeled as being linearly proportional to the abundance of that subpopulation^5^,^6^ (without log transformation^16^, Fig. 1c). Because many genes can be co-expressed across different subpopulations, CAM instead identifies the marker genes by detecting the simplex vertices of mixed expression data. The minimum description length (MDL) criterion determines the number of subpopulations present^17^ (Methods).

## Results

### Validation of CAM on real benchmark dataset (GSE19830)

**Figure 1:**
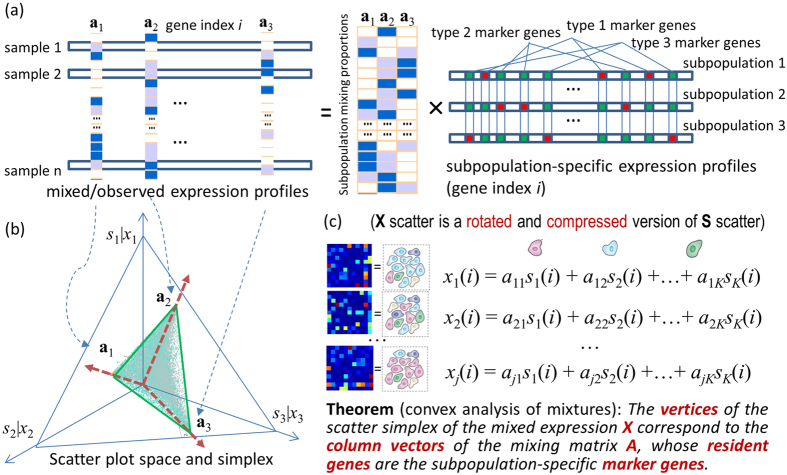
CAM principles for unsupervised identification of novel subpopulation-specific marker genes. Marker genes are the genes whose expressions are exclusively enriched in a particular subpopulation, where *s*_*k*_(*i*) is the expression level of gene *i* in subpopulation *k*, *x*_*j*_(*i*) is the expression level of gene *i* in heterogeneous sample *j*, and *a*_*jk*_ is the proportion of subpopulation *k* in heterogeneous sample *j*. In the linear latent variable modeling, mRNA expression levels from a mixture of multiple subpopulations are modeled as the weighted average of the expression levels from a set of distinct subpopulations, where the weights describe the proportions of each distinct subpopulation in the overall population. (**a**) Linear latent variable modeling of mixed gene expression data in which the subpopulation-specific marker genes explicitly preserve the information on the mixing proportions of all the subpopulations present in the heterogeneous samples. (**b**) Geometry of the mixing operation in scatter space that produces a compressed and rotated scatter simplex whose vertices host subpopulation-specific marker genes and correspond to mixing proportions. (**c**) Mathematical description on the gene expression readout of multiple distinct subpopulations and the key theorem on the geometric identifiability of subpopulation-specific marker genes from mixed expression data.

**Figure 2:**
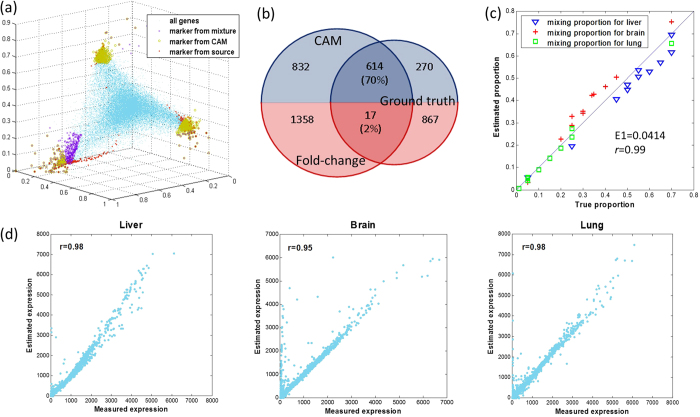
Validation of CAM for blindly identifying subpopulation-specific marker genes (distinct subpopulations include liver, lung, brain). (**a**) Identification of marker genes by CAM from biologically mixed gene expression data (Blue dots: all genes forming scatter simplex. Red diamonds: ground truth marker genes extracted from subpopulation expressions. Brown circles: marker genes identified by CAM from mixed expression data. Pink crosses: marker genes identified by standard differential analysis from mixed expressions.). (**b**) Venn diagram showing the marker genes detected by CAM and simple fold-change, respectively, against the ground truth, with sufficiently higher sensitivity (70%) and specificity (>90%) offered by CAM. (**c**) Scatter plot of the estimated subpopulation proportions across heterogeneous samples by CAM, plotted against the ground truth, with an almost perfect correlation coefficient. (**d**) Scatter plots of the estimated subpopulation-specific gene expression profiles deconvoluted from heterogeneous tissue samples, plotted against the measured gene expression profile from pure subpopulations, with an almost perfect correlation coefficient calculated over only the marker genes.

Unsupervised identification of marker genes from mixed expression data allows us to acquire the relative expression levels of those genes (Fig. 1a). The average of sum-normalized marker gene expressions produces subpopulation proportions (Methods and Fig. 1b). Using predesigned RNA mixing experimental data acquired from biological mixtures of pure gene expressions (brain, liver, lung)^5^,^6^, we showed that CAM identified the marker genes that define each of the multiple subpopulations (Fig. 2a,b) and estimated the proportions of these subpopulations in the mixed samples (Fig. 2c) and their respective expression profiles (Fig. 2d). Since the presence of marker genes is both a sufficient and necessary condition for deconvolution (Methods), these results (validated by the ground truth) confirm the existence of marker genes and CAM’s ability to detect these genes blindly and correctly (Fig.2b). Moreover, CAM enabled detection of condition-specific marker genes across sample groups (for example, disease versus control). Thus, novel marker genes for a subpopulation in a given context can be determined, despite an expected change in that subpopulation’s relative abundance and/or state.

**Figure 3:**
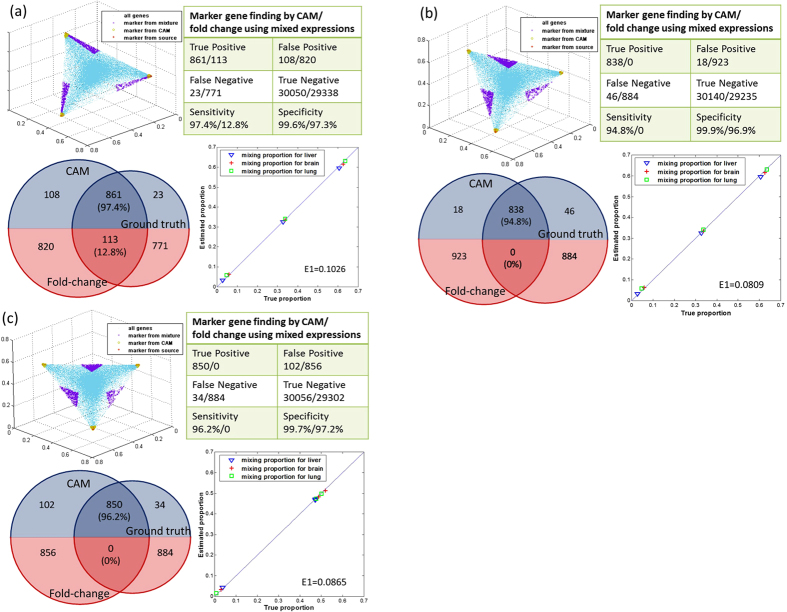
CAM validation on synthetic GSE19830. The marker genes identified by CAM were validated and compared with the results of a standard differential analysis using mixed expressions. Mixed expression values were reconstituted by multiplying the measured pure subpopulation expressions (brain, liver and lung) by the proportions of subpopulations in a given heterogeneous sample, corresponding to 30^°^, 45^°^ and 60^°^ simplex rotations and an arbitrary compression. A total of 884 marker genes were identified using one-*vs*-each fold change on pure expression data, which served as the ground truth^1^,^7^,^37^. (**a**) Validation results on 30^0^ rotation: scatter simplex, sensitivity and specificity, Venn diagram (blue part shows the marker genes blindly detected by CAM using mixed expression data versus those detected by fold change using source tissues; red part shows the marker genes detected by fold change using mixed expressions versus those detected using source tissues), and proportion estimates. (**b**) Validation results on 45^0^ rotation: scatter simplex, sensitivity and specificity, Venn diagram, and proportion estimates. (**c**) Validation results on 60^0^ rotation: scatter simplex, sensitivity and specificity, Venn diagram, and proportion estimates.

We then validated and compared the marker genes identified by CAM with the results of a standard differential analysis using mixed expressions, in a situation in which all ground truths are known. We reconstituted mixed expressions by multiplying the measured pure expressions (brain, liver, lung) by the proportions of subpopulations in a given heterogeneous sample. Such mixtures mimic biological samples with varying relative abundances of different subpopulations. Experimental results on three different mixture settings (Fig. 3a–c corresponding to 30^°^, 45^°^ and 60^°^ simplex rotations) show that CAM identified a significant majority (>90%) of ground truth marker genes that were largely missed by standard differential analysis (Fig. 3a–c). Moreover, the overall constituent proportions estimated by CAM correlate well with the ground truth (*r* > 0.95; Supplementary Table S1). We also analyzed the same datasets with supervised methods and showed their instability in the presence of uncertainly measured cell proportions or signatures^5^,^6^, referring to the significantly degraded performance when the required supervising information is inaccurate or unreliable (Supplementary Figs. S1 and S2, Supplementary Discussion).

### Validation of CAM on real benchmark dataset (GSE11058)

In most physiologically relevant settings, few universal marker genes are available for all subpopulations in a tissue sample, particularly under different conditions. Furthermore, no ‘gold-standard’ marker gene list exists to accurately define many of the known subpopulations that may be present in a sample. To test CAM’s ability to detect novel marker genes in complex tissues, we applied CAM to the benchmark human blood gene expression data^11^. In this dataset, the constituent subpopulations are four immune cells in blood that are phenotypically very similar to each other: Raji (B-cell), IM-9 (B-cell), Jurkat (T-cell), and THP-1 (monocyte). The expression profiles of the purified subpopulations were not used in any step of CAM but served as the ground truth for performance assessment.

**Figure 4:**
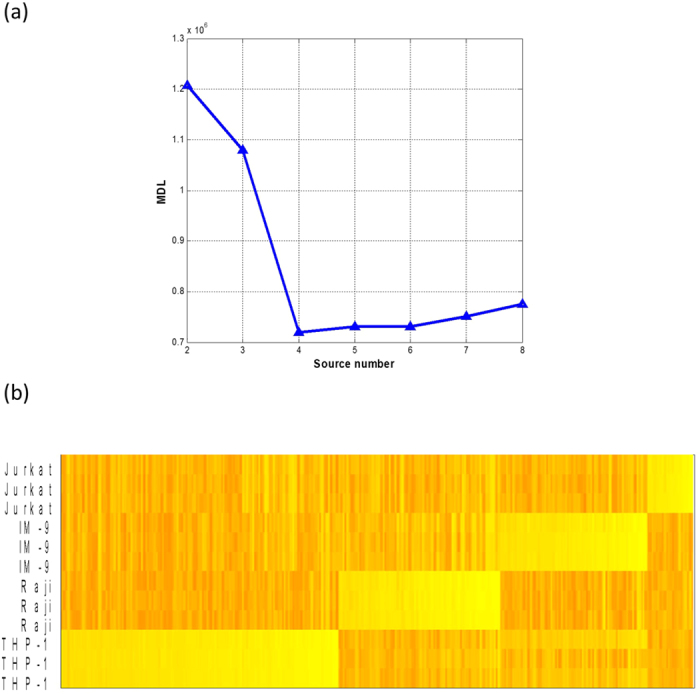
CAM validation on GSE11058^11^ – blindly detected subpopulation -specific marker genes (301) and MDL curve for detecting the number of sources (*e.g.*, subpopulations). The mixtures were obtained from biologically mixed expression profiles of four subpopulations, *i.e*., transformed cell lines of immune origin: Raji and IM-9 (both from B cells), Jurkat (from T cells), and THP-1 (from monocyte) cells. Each of four pure cell lines has three duplicates. (**a**) The MDL values are calculated over *K* = 2, 3, …, 8, with the minimum value corresponding to *K* = 4. (**b**) The heat map of 301 marker genes that are blindly detected by CAM.

**Figure 5:**
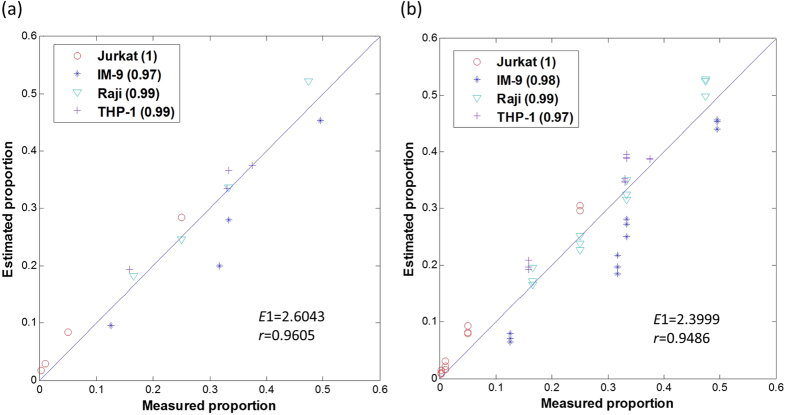
CAM validation on GSE11058 11 – proportion estimates (GT stands for ground truth). (**a**) Scatter plot of the true and estimated proportions of four cell lines in the 4 mixture samples (average profile of 3 replicates). (**b**) Scatter plot of the sample-specific true and estimated proportions of four cell lines in the original 12 mixture samples.

Without using any *a priori* information, the minimum value of the MDL curve correctly indicated the presence of four subpopulations in the mixtures (Fig. 4a); CAM blindly identified 301 marker genes (Fig. 4b) and accurately estimated both the constituent proportions (*r* = 0.96; Fig. 5, Supplementary Table S2) and the subpopulation-specific expression profiles (*r* = 0.95~0.98; Supplementary Fig. S3). CAM even slightly outperformed the supervised method that used the known reference marker gene signatures (Supplementary Table S3). These results further support the existence of novel marker genes and CAM’s ability to detect them both blindly and effectively. Note that the correlation between the estimated and measured pure expression profiles is assessed over marker genes rather than all genes since the high correlation obtained using all genes would be misleading due to the presence of many co-expressed genes (Supplementary Table S4). We next assessed the biological plausibility of the 301 blindly detected marker genes. Analysis with Ingenuity Pathway Analysis (IPA) shows that these novel marker genes are enriched in functions associated with each of the four subpopulations (Supplementary Fig. S4, Supplementary Discussion).

### Application of CAM on real benchmark dataset (yeast cell cycle)

**Figure 6:**
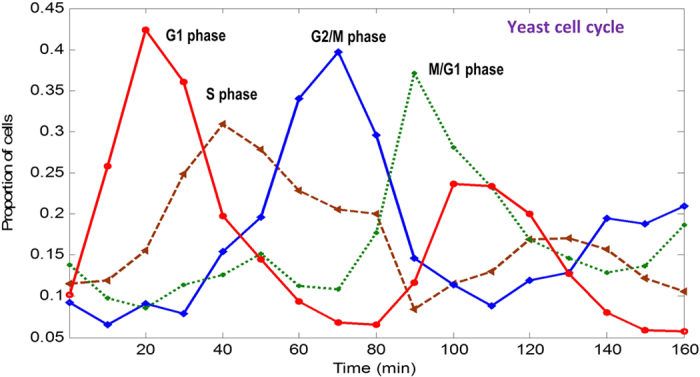
Application of CAM for blindly identifying subpopulation-specific marker genes (distinct subpopulations include yeast cells at four different cell-cycle states). From the gene expression time-courses of a synchronized yeast cell population, CAM detected cell state-specific marker genes and revealed cell-cycle repopulation dynamics that were undetectable at heterogeneous population level, where the estimated proportion of cells in each cell cycle phase, plotted as a function of time, match well with the phases observed by microscopy and FACS analysis.

Interactions among cells are fundamental to many biological processes including repopulation dynamics^4^. As a more challenging case involving only a single cell type, we studied yeast grown under varying conditions to reinterpret cell cycle dynamics on the basis of their gene expression changes over time^10^,^18^. The mixed expressions were acquired from synchronously growing yeast cells in different phases of the cell cycle, where cells exist at different phases even within a given cell type, presenting an additional layer of complexity to identify differentiation marker genes. In marked contrast to supervised methods that require *a priori* information, CAM automatically detected phase-specific marker genes of yeast cells and uncovered the subpopulation dynamics (cell cycle phase distributions) (Fig. 6). These results correlate well with the patterns reported by a supervised method in the same dataset^10^. We also assessed these results by comparing with the phase-specific patterns generated by the marker genes of Spellman *et al.*^18^ and obtained high accordance (Supplementary Fig. S5, Supplementary Discussion). Gene enrichment analysis with DAVID confirmed the biological plausibility of the 187 phase-specific marker genes blindly identified by CAM^19^ (Supplementary Table S5, Supplementary Discussion).

## Discussion

**Figure 7:**
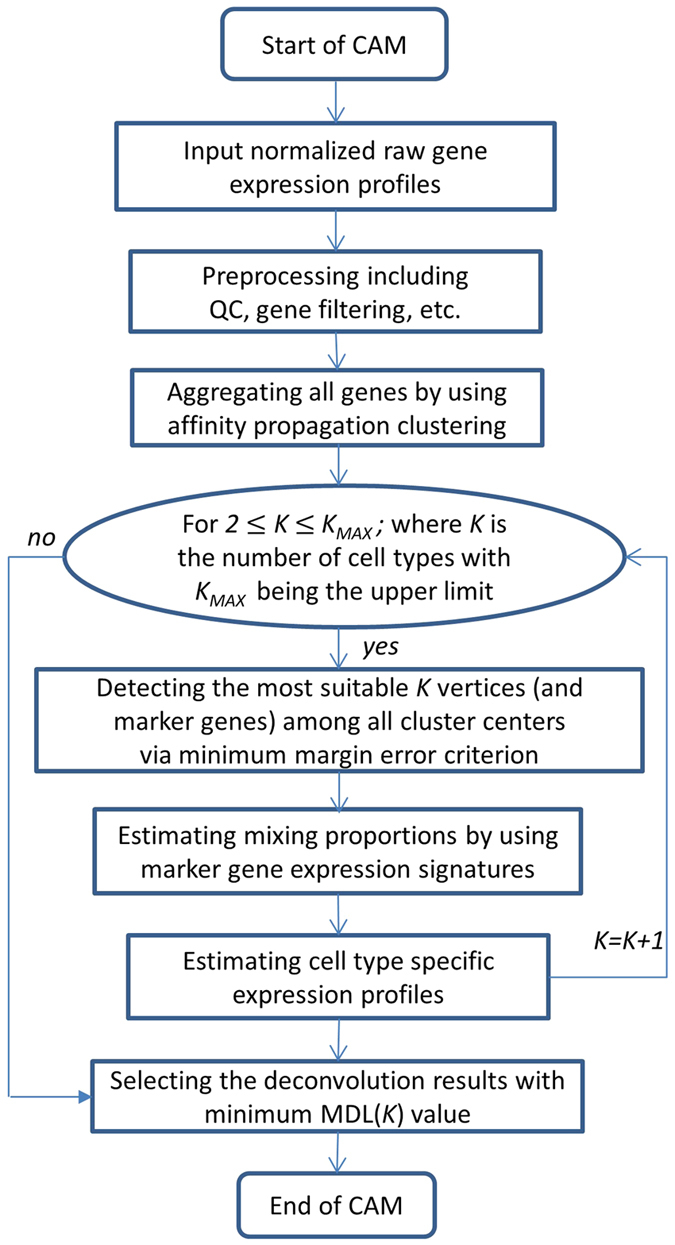
Overall flowchart of the CAM algorithm.

Most of the large datasets available for study in many diseases are based on the use of complex tissue samples with unknown subpopulations and where no supervising information is available^12^,^20^. Currently, CAM provides the only validated tool to identify marker genes and to resolve tissue heterogeneity in these public datasets (Fig. 7). The significant advantage of our strategy is its ability to detect novel marker genes in an unsupervised way using the data obtained from complex tissue samples. Importantly, CAM operates in the scatter space of the mixtures, and hence does not require the presence of pure-subpopulation samples (phenotypic archetypes)^14^,^15^. This advantage is significant in that CAM can achieve all of its goals using only a small number of heterogeneous samples (when the number of heterogeneous samples equals or is greater than the expected number of distinctive subpopulations), and is more powerful to distinguish between phenotypically similar subpopulations (leveraging the mixing diversity across heterogeneous samples). Supported by the MDL model selection, the benefits of CAM method include its wide and unbiased applicability, and exciting potential for characterizing subpopulations in tissue remodeling (Supplementary Discussion). While the principal application here involves gene expression data, our methodology can be readily applied to other types of quantitative measurements^21^. We have applied CAM to unmixing other types of data such as medical imaging and remote sensing^22^,^23^,^24^,^25^,^26^ and obtained very promising results.

The CAM method is principally applicable to the situation when the number of available samples is less than the number of subpopulations, *i.e.*, an underdetermined problem, albeit with some flexibility on model assumptions^27^. However, in such situation, while the CAM solution will still be able to identify the mixing matrix (including the most appropriate number of sources), the subpopulation-specific gene expression profiles cannot be accurately estimated^27^.

Supervised methods are poorly suited to finding subpopulations that are subtle, condition-specific (their distinctive signatures are changed when the cells are present in different microenvironments or tissues), or previously unknown. Unsupervised approaches have not previously been reported, and yet these are necessary and urgently needed for appropriate data analysis. To provide some perspective, adequate information needed to guide supervised analyses is lacking in most of the datasets within large international studies and databases such as TCGA, METABRIC, GEO, ONCOMINE and many others.

Alternative approach by Schwartz *et al.*^14^ and by Hart *et al.*^15^, which may be the closest to our approach, was not designed to address the same problems as ours and it is not an unsupervised approach. This method operates in sample space, requires the presence of pure subpopulation samples (phenotypic archetypes), and uses a supervised/subjective scheme to determine the number of constituent subpopulations^15^. The differences (and consequent limitations) are highly significant. Regarding the key difference between the CAM here and alternative methods, we should emphasize that our CAM method operates in scatter space and requires only a limited number of subpopulation-specific markers^23^,^24^. In scatter-space the power of detecting simplex vertices solely depends on the non-singularity (diversity) of the mixing proportions yet is independent of phenotypic similarity among subpopulations. In contrast, in the sample-space approaches, pure subpopulation samples are required for each of the subpopulations. While as assumed by Schwartz *et al.* that pure subpopulation samples may be available in the limit of uniform, dense sampling^14^, this assumption is often invalid when sample size is relatively small, and often close phenotypic similarity between subpopulations will be a problem in identifying the vertices in sample space. These issues could greatly limit the applicability of the sample-space based unsupervised methods.

In relation to previous work, while this is the first report on CAM methodology with specific designs and applications to identifying novel markers and subpopulations in complex tissues using gene expression data, the concept of exploiting the convex analysis for general unsupervised deconvolution is shared by prior publications^25^. For example, sample space or compartment model based method has been applied to analyzing remote sensing or medical imaging data^22^,^24^,^26^,^28^, facilitated by the open-source software tool^23^,^29^. In contrast, the more complete mathematical derivations and CAM algorithm, *e.g.*, the MDL formulation, marker gene concept, and theorem proofs, are specifically derived for analyzing gene expression data in biological tissue samples.

## Methods

### Latent variable model on mixed gene expressions in heterogeneous samples

Consider gene expression measured from a sample composed of *K* subpopulations. We assume that the measured expression level *x* is the weighted sum of each subpopulation’s expression, where the contribution from a single subpopulation is proportional to the abundance and specific expression of that subpopulation^2^,^5^,^6^,^10^,^16^. The measured expression level thus is (Fig. 1c)


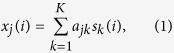


where *s*_*k*_(*i*) is the expression level of gene *i* in subpopulation *k*, *x*_*j*_(*i*) is the expression level of gene *i* in heterogeneous sample *j*, and *a*_*jk*_ is the proportion of subpopulation *k* in heterogeneous sample *j*. We further assume that gene expression values are non-negative (before log-transformation^13^,^16^) and adopt the definition of subpopulation-specific marker genes as those genes whose expression values are exclusively enriched in a particular subpopulation^6^,^10^,^11^ (Fig. 1a). Thus, the specific expression of a marker gene (MG) in subpopulation k* is


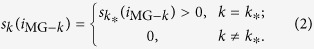


When marker genes are known for each subpopulation, we can use the expression values of marker genes to deconvolute mixed expression profiles^6^,^10^,^11^. When no such prior knowledge is available (*i.e*., none of *K*, *a*_*jk*_ and *s*_*k*_(*i*_MG_) is known *a priori*), solving latent variable model (eq. (1)) is essentially a blind source separation problem^22^,^25^, where accurate identification of subpopulation-specific marker genes is a critical but nontrivial task^6^,^10^,^20^.

Our formulation dissects complex transcriptional heterogeneity into combinations of distinct subpopulations, leveraging the advantages of both tissue-wide and single-cell approaches^14^,^30^. Specifically, discerning differences among single cells can gain valuable information about inter-cellular heterogeneity but allow only a few markers per cell and is prone to cell-cycle confounders; while tissue-wide measures provide a detailed picture of averaged population state but at the cost of losing information about inter-subpopulation heterogeneity.

### Parallelism between latent variable model and the theory of convex sets

Consider a set of *J* (≥*K*) heterogeneous samples of varying composition of unknown subpopulations. Applying a sum-based standardization to gene expression values *x*_*j*_(*i*) across samples and using vector-matrix notation, we can re-express equation (1) as


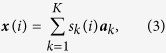


where ***x***(*i*) and ***a***_*k*_ are the vector notations (over samples) of mixed expression values and subpopulation proportions, respectively. Since *s*_*k*_(*i*) is non-negative and standardized, as a non-negative linear combination of 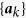, the set of gene expression vectors ***x***(*i*) forms a subset of the *convex set* uniquely defined by the set of 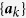
25,26,31 (Fig. 1b)


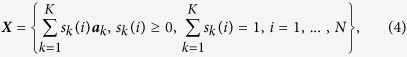


where *N* is the number of genes.

### Mathematical foundation for unsupervised identification of novel marker genes

We propose the Convex Analysis of Mixtures (CAM) framework to exploit the strong parallelism between a linear latent variable model (eq. (3)) and the theory of convex sets. The novel insight is that subpopulation-specific marker genes that define latent pure subpopulations reside at the extremities of the scatter simplex formed by all genes, while the interior of the simplex is occupied by co-expressed genes (whose values are linear non-negative combinations of pure subpopulation expression values) (Fig. 1b). We can then identify novel marker genes by geometrically locating the vertices of the multifaceted simplex that most tightly encloses the gene expression profiles and has the same number of subpopulations as vertices. CAM is supported theoretically by a well-grounded mathematical framework as summarised in the following newly proven theorems (see formal proofs in Supplementary Method).

### Lemma 1 (Scatter compression and rotation)

Suppose that pure subpopulation expressions are non-negative, and **x**(i) = **a**_1_s_1_(i) + … + **a**_k_s_k_(i) + … + **a**_K_s_K_(i) where **a**_k_’s are linearly independent and non-negative, then, the scatter simplex of pure subpopulation expressions is compressed and rotated to form the scatter simplex of mixed expressions whose vertices coincide with **a**_k_’s.

The terms ‘compressed and rotated’ are used here to illustrate the geometric transformation on the simplex made by the mixing process in scatter space. Given the fact that in this case the proportions ***a***_***k***_’s are also non-negative (in addition to gene expressions being non-negative), as the result, Lemma 1 shows that every mixed gene expression data point ***x***(*i*) is confined within the simplex (convex hull) defined by ***a***_***k***_’s. Since ***a***_***k***_’s are resided within the first quadrant while the simplex of pure subpopulation expressions ***s***(*i*) originally occupy the first quadrant, we therefore use the terms ‘compressed and rotated’ to describe such transformation.

### Theorem 1 (Unsupervised identifiability)

Suppose that pure subpopulation expressions are non-negative and subpopulation-specific marker genes exist for each constituting subpopulation, and **x**(i) = **a**_1_s_1_(i) + … + **a**_k_s_k_(i) + … + **a**_K_s_K_(i) where **a**_k_’s are linearly independent and non-negative, then, the vertices of the scatter simplex of mixed expressions host subpopulation-specific marker genes and coincide with **a**_k_’s that can be readily estimated from marker gene expression values with appropriate rescaling.

From Lemma 1 and Theorem 1, there is a feasible mathematical solution to identify subpopulation-specific marker genes from the measured gene expression mixtures: in principle, under a noise-free scenario, we can blindly identify novel marker gene indices by locating the *vertices* of the mixed expression scatter simplex^22^,^23^,^24^. We emphasize that CAM can distinguish between phenotypically similar subpopulations, by working in scatter space in which the power of detecting simplex vertices depends solely on the mixture diversity (a basic requirement for any inverse problem) rather than phenotypic diversity^14^. This is significantly different from the relevant approaches (including our own other previous work) that operate in sample space and require the presence of pure subpopulation samples^14^,^15^,^25^.

### Data preprocessing

First, we eliminate genes whose signal intensity (vector norm) is lower than 5% (noise) or higher than 95% (outlier) of the mean value over all genes. The signals from these genes are unreliable and could have a negative impact on the subsequent analyses (Supplementary Method). Second, when *J *≫ *K*, dimension reduction is performed on the raw measurements using principal component analysis, sample clustering or nonnegative matrix factorization techniques, to improve the efficiency of subsequent analyses^13^,^23^.

### Aggregation of gene expression vectors

To further reduce the impact of noise/outlier data points (gene expression vectors over samples), improve the efficiency of CAM, and permit appropriate parameterization of the MDL criterion to determine the number of subpopulations, we aggregate gene vectors into representative clusters using affinity propagation clustering (APC)^23^,^24^,^26^,^32^ (Supplementary Method). As an initialization-free and near-global-optimum clustering method, APC simultaneously considers all gene vectors as potential exemplars and recursively exchanges real-valued ‘messages’ between gene vectors until a high-quality set of exemplars and corresponding clusters gradually emerge. The APC algorithm is data-driven, so the message-passing procedure may be terminated after a fixed number of iterations or after the updates stay constant for some number of iterations. In all of our experiments, we adopted a default damping factor of 0.5. The update rules are repeated iteratively and terminated when no further change occurs for about 10 iterations^23^,^24^. Our experience indicates that these default algorithmic parameter settings are quite suitable for obtaining good results.

### Convex analysis of mixtures (CAM)

To identify the *vertices* of clustered convex set ***X*** (scatter simplex of mixed expression profiles), we performed CAM on the obtained *M* cluster centers 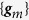 of gene vectors. We assumed *K* true vertices and conducted an exhaustive combinatorial search (with total 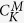 combinations), based on a convex-hull-to-data fitting criterion, to identify the most probable *K* vertices. We used the margin-of-error


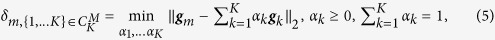


to quantify the distance between 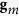 and convex set ***X*** defined by 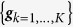, where we have 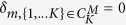 if 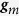 is inside ***X***. We then selected the most probable *K* vertices when the corresponding sum of the margin-of-error between the convex hull and the remaining “exterior” cluster centers reaches its minimum^22^,^23^,^26^:


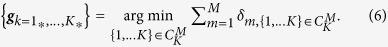


Subsequently, we identified the indices of subpopulation-specific marker genes based on the memberships associated with 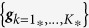, where 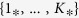 denote the cluster indices of the true simplex vertices, and the genes assigned to gene cluster at a vertex 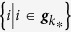 are declared to be marker genes, *i.e.*, 
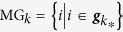
.

### Model selection procedure

One important discovery step for CAM (as a fully unsupervised method) is to automatically detect the number *K* of cell subpopulations in the heterogeneous samples. We used MDL, a widely-adopted and consistent information theoretic criterion^17^, to guide model selection^26^ (Supplementary Method). We performed CAM on several competing candidates, and selected the optimal model that assigns high probabilities to the observed data with parameters that are not too complex to encode^17^. Specifically, a model is selected with *K* subpopulations by minimizing the total description code length defined by^26^


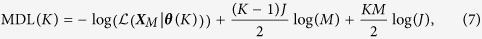


where denotes the joint likelihood function of the clustered latent variable model, ***X***_*M*_ denotes the set of *M* gene vector cluster centers, and 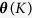 denotes the set of freely adjustable parameters in the clustered latent variable model^23^,^24^,^26^. Moreover, when estimating the mixing matrix (*i.e.*, the column vectors ***a***_*k*_*’s*) that is parameterized by (*K*-1)*J* independent entries, we use some form of vector-average operation (*i.e.*, 
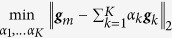
), where the scalar entry *a*_*k*_(*j*) is estimated from *M* scalar entries *g*_*m*_(*j*) for a given *j*, contributing total (*K*-1)*J*log(*M*)/2 bits. Similarly, when estimating the subpopulation-specific gene expression profiles (the row vectors ***s***_*k*_) with total *KM* entries, we use a vector-average operation (*i.e.*, solving linear equations), where the scalar entry *s*_*k*_(*m*) is estimated involving only *J* scalar entries *g*_*m*_(*j*) for a given *m*, contributing total *KM*log(*J*)/2 bits.

### Estimation of constituent proportions and subpopulation-specific expression profiles

On the basis of the expression levels of subpopulation-specific marker genes detected by CAM, the relative proportions of constituent subpopulations are estimated using standardized averaging,


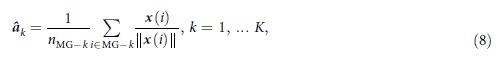


where MG-*k* is the index set of marker genes for subpopulation *k*; *n*_MG-*k*_ is the number of marker genes for subpopulation *k*; and ||.|| denotes the vector norm (*L*_*1*_ or *L*_*2*_). The resulting 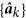 are then used to deconvolute the mixed expressions into subpopulation-specific profiles by non-negative least-square regression techniques^6^,^10^,^11^,^23^,^26^.

### Performance evaluation criteria

We use four complementary evaluation criteria and the ground truth to assess the performance of CAM. To assess the membership match and mismatch between the subpopulation-specific marker genes detected from pure subpopulation expressions versus from original mixed expressions, we use Venn diagrams to visualize the outcomes and also calculate both sensitivity and specificity. To assess the accuracy of subpopulation proportion estimates 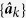, in addition to classic Pearson correlation coefficient 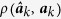, we also adopt the *E*1 criterion given by^33^


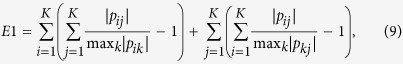


where *p*_*ij*_ is the *ij*th element of the matrix 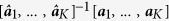. Note that *E*1 is invariant to permutation or scaling, and *E*1 = 0 when the estimation is perfect. Moreover, to assess the accuracy of estimated subpopulation-specific expression profiles 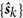, we calculate Pearson correlation coefficient between the estimated expression profile and ground truth 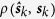 over ‘subpopulation-specific marker genes’ and ‘all genes’, respectively.

### Robust detection of marker genes on numerically mixed expressions (synthetic data)

To validate CAM’s ability to blindly detect marker genes from mixed gene expression profiles, we numerically generated three different sets of mixtures using the profiles of pure liver, brain and lung tissues^5^, corresponding to 30^o^, 45^o^ and 60^o^ simplex rotations and arbitrary compressions (Fig. 3). We performed standard differential analysis (one-versus-everyone^7^) on the gene expression profiles of the three source tissues to construct a ‘gold standard’ set of 884 marker genes. We applied CAM to these mixtures and compared the marker genes blindly detected by CAM to the gold standard set, assessed by both sensitivity and specificity. In these experiments, we set *K* = 3 as supported by MDL based model selection. Using these blindly detected marker genes, we estimated the constituent proportions in each of the mixtures and subpopulation-specific expression profiles (Fig. 3, Supplementary Table S2).

### Performance comparison between CAM and supervised methods (synthetic data)

To compare the performance between unsupervised CAM and supervised methods, we analyzed the same benchmark datasets with supervised methods^5^,^6^. Since *a priori* information used in supervised methods is prone to various errors and is often condition-specific, we mainly assessed the reliability of these methods by numerically perturbing either the baseline constituent proportions^5^ or the reference signatures^6^. We first randomly perturbed the constituent proportions with ± 0.05 ∼ 0.3 or replaced one of the mixtures with arbitrary constituent proportions, and compared the estimated subpopulation-specific profiles against ground truth using correlation coefficient over marker genes^5^. We next randomly perturbed the reference signatures with ± 1 ∼ 50% expression variations or replaced some of the marker genes with arbitrary ones, and compared the estimated constituent proportions against ground truth via correlation coefficient^6^. These comparison experiments were performed on a large number of replications (Supplementary Figs. S1–S2, Supplementary Tables S6–S7, Supplementary Discussion).

### Accurate detection of marker genes on biologically mixed expressions (GSE19830)

To further validate CAM’s ability to blindly detect marker genes from mixed gene expression profiles, we analyzed the benchmark real gene expression dataset GSE19830^5^. The dataset contains 3 source tissue samples and 11 biologically mixed heterogeneous samples with known mixing proportions, with each sample having 3 replicates^5^ (Supplementary Table S6). Using the marker genes blindly detected by CAM, we estimated the constituent proportions in each heterogeneous samples and subpopulation-specific expression profiles (Fig. 2a–d, Supplementary Fig. S6). Besides the Venn diagrams comparing the marker genes detected by CAM with the gold standard (Fig. 2b), we also assumed the coexistence of distinct subpopulations and subpopulation-specific marker genes, since a distinct subpopulation would logically require a set of marker genes that are ‘unique’ to that subpopulation. In our newly proved two theorems, the existence of subpopulation-specific marker genes is both a sufficient and a necessary condition for a successful blind deconvolution. Thus, if a successful deconvolution of mixed expression data is achieved, the existence of subpopulation-specific marker genes is almost certain (Supplementary Discussion).

### Effective detection of marker genes on biologically mixed expressions (GSE19380)

We then applied CAM to the benchmark real gene expression dataset GSE19380^6^. The three rat primary subpopulations (neuron, astrocytes, oligodendrocytes) were cultured separately, and their mRNAs were extracted and variably mixed to generate gene expression data using Affymetrix Rat 230 2.0. Without using any prior information, CAM blindly detected 183 probe sets that are located at the scatter simplex vertices of mixed expression profiles. These probe sets were confirmed by the classic analysis using the pure subpopulation expression profiles. We accurately estimated the subpopulation proportions with a correlation coefficient of 0.99, and the subpopulation-specific gene expression profiles with a correlation coefficient of 0.94–0.99, when compared with the ground truth. We found that while *a priori* marker genes (Supplementary Table S7) used by supervised method^6^ were near the vertices of pure subpopulation expression scatter simplex, some of these *a priori* marker genes clearly deviated from the vertices of the mixed expression scatter simplex.

### Analyses of benchmark blood cell mixed expressions (GSE11058)

The dataset contains 24 samples of four subpopulations, each with three replicated gene expression profiles of variable combinations^11^ (Supplementary Table S2). We averaged the three replicates on each of 12 pure and 12 mixed expression profiles, resulting in 4 pure subpopulation and 4 mixed gene expression profiles. After pre-processing and normalization by MAS 5.0, total 13,000 probe sets were retained. The 301 novel marker genes blindly identified by CAM from mixed expressions meet almost perfectly to the definition of marker genes, i.e., genes whose expressions are exclusively enriched in a particular subpopulation and functionally enriched^6^,^7^ (Supplementary Fig. S4). We also compared the deconvolution results of CAM and supervised methods^11^,^12^,^13^ (Supplementary Tables S3 and Table S4f). This is particularly encouraging, since it means that novel marker genes for phenotypically similar subpopulations, given sufficient mixture diversity, can be detected efficiently by CAM.

### Analyses of benchmark yeast cell cycle time-course data (CDC28)

Cells were collected at 17 time points taken at 10 minute intervals, covering nearly two full cell cycles of synchronized yeast cell growth under variable temperatures^10^. The gene expression profiles contain 6208 transcripts across 4 discrete phases^34^. Note that here CAM exploited the temporal diversity to perform the unsupervised analysis. CAM identified total 187 phase-specific marker genes^34^,^35^, among which 114 (61%) are previously reported^18^,^36^ and 73 (39%) are considered novel, with 22 genes (21.2%) being verified cell cycle regulated genes^35^,^36^. Using DAVID to explore gene set functions, we found that 21 genes are enriched in G1 phase, 58 in S phase, 16 in G2/M phase, and 68 in M/G1 phase (Supplementary Table S5). The repopulation dynamics curves revealed blindly by CAM (*K* = 4 suggested by MDL, Supplementary Fig. S7) are highly consistent with the known cell cycle patterns^18^ (Supplementary Fig. S5, Supplementary Discussion). This is particularly appealing in that when a sufficient number of diversely-mixed samples is available, CAM should uncover all subpopulations of different cell types and identify the same cell type at different cell-cycle phases^30^.

### Availability of CAM software and supporting data

A Java-R package of CAM is available for academic and non-commercial use at: http://mloss.org/software/view/437. In addition, public gene expression data analyzed in this paper are also available from the Gene Expression Omnibus Database under Accession Number GEO: GSE19830, GSE19380, GSE11058, and CDC28.

## Additional Information

**How to cite this article**: Wang, N. *et al.* Mathematical modelling of transcriptional heterogeneity identifies novel markers and subpopulations in complex tissues. *Sci. Rep.* **6**, 18909; doi: 10.1038/srep18909 (2016).

## Supplementary Material

Supplementary Information
