# Supplementary material for: Mathematical modelling of transcriptional heterogeneity identifies novel markers and subpopulations in complex tissues
Source: Sci Rep. 2016 Jan 7;6:18909. doi: 10.1038/srep18909 (PMC4703969; doi:10.1038/srep18909)
Supplement: Supplementary Information [file srep18909-s1.pdf]

## Supplementary Figures & Tables

# Mathematical modelling of transcriptional heterogeneity identifies novel markers and subpopulations in complex tissues

Niya Wang, Eric P. Hoffman, Lulu Chen, Li Chen, Zhen Zhang, Chunyu Liu, Guoqiang Yu, David M. Herrington, Robert Clark, and Yue Wang


| | |
|---|---|
| **Supplementary Figure 1** | Unreliability of supervised method on GSE19830 |
| **Supplementary Figure 2** | Unreliability of supervised method on GSE19380 |
| **Supplementary Figure 3** | CAM validation on GSE11058 – subpopulation-specific profiles |
| **Supplementary Figure 4** | IPA on CAM detected marker genes on GSE11058 |
| **Supplementary Figure 5** | CAM validation on cell cycle data – comparison with peers |
| **Supplementary Figure 6** | CAM validation on GSE19830 – MDL curve |
| **Supplementary Figure 7** | CAM validation on cell cycle data - MDL curve |
| | |
| **Supplementary Table 1** | CAM validation on GSE19830 – proportion estimates (A) |
| **Supplementary Table 2** | CAM validation on GSE11058 – proportion estimates |
| **Supplementary Table 3** | CAM validation on GSE11058 – comparison with peers |
| **Supplementary Table 4** | Misleading assessment using all genes – on GSE11058 |
| **Supplementary Table 5** | CAM Marker Gene Enrichment Analysis on cell cycle data |
| **Supplementary Table 6** | CAM validation on GSE19830 – proportion estimates (B) |
| **Supplementary Table 7** | *A priori* marker gene list used by Kuhn et al. on GSE19380 |

Supplementary Figure 1a-f.

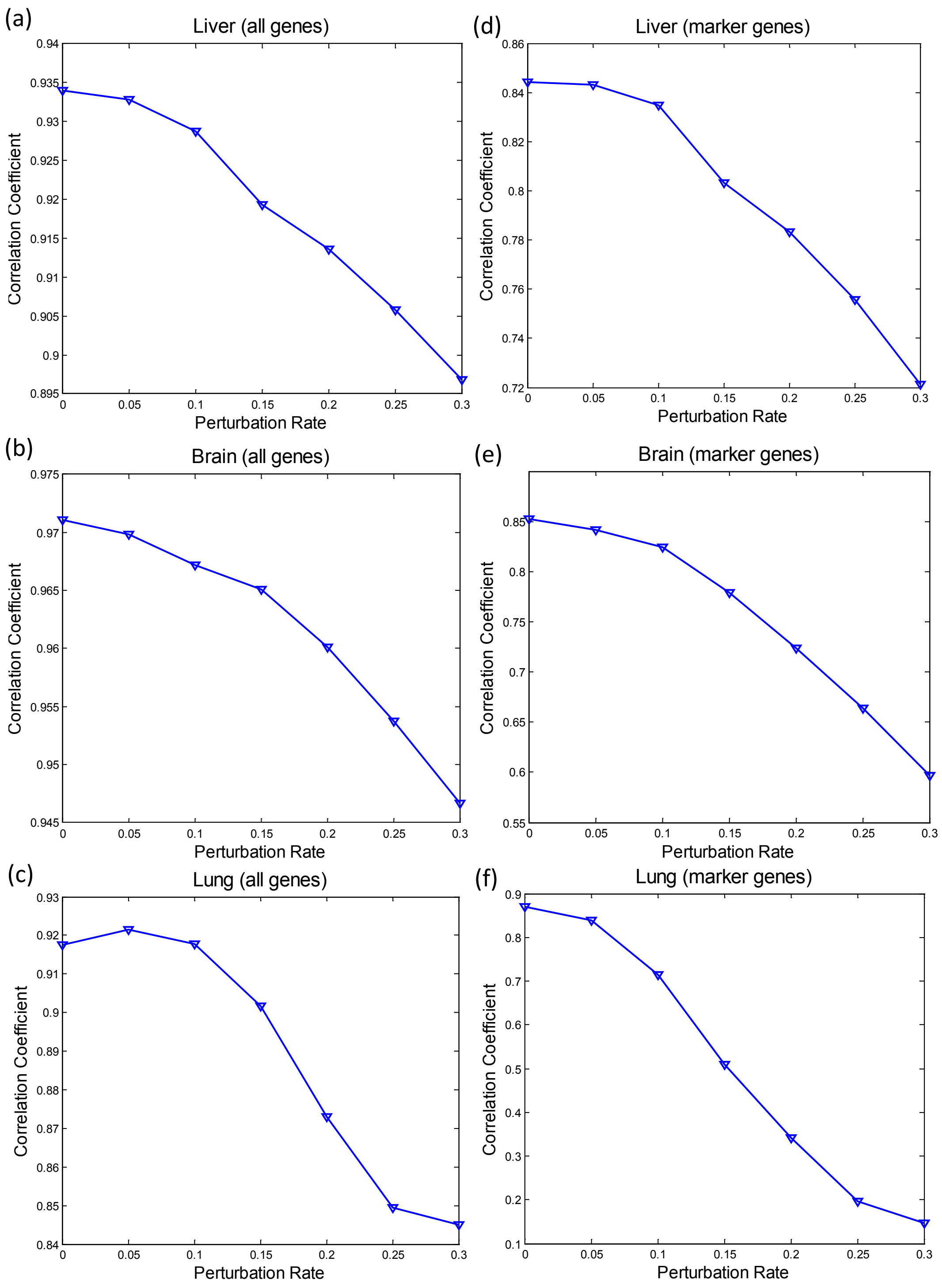

# Supplementary Figure 1g-l.

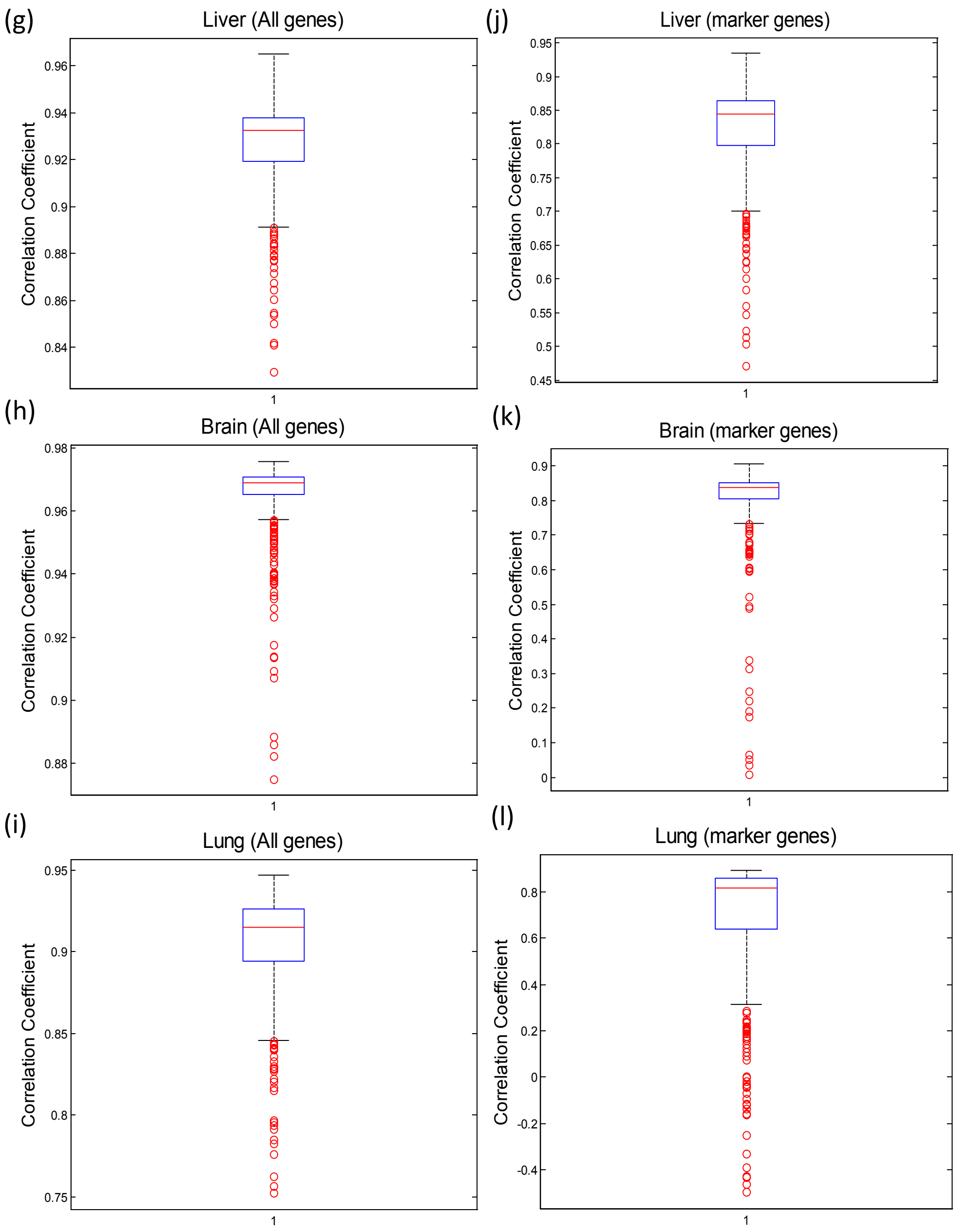

Supplementary Figure 2.

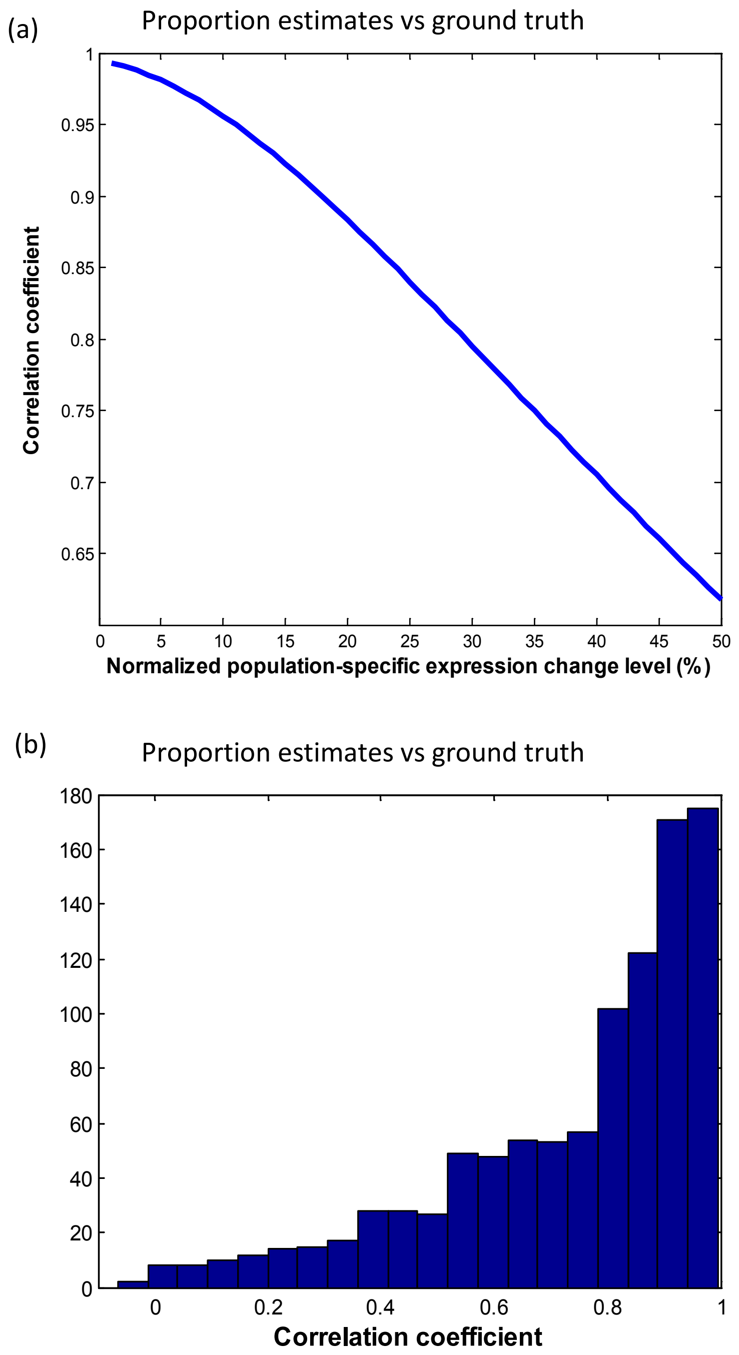

Supplementary Figure 3.

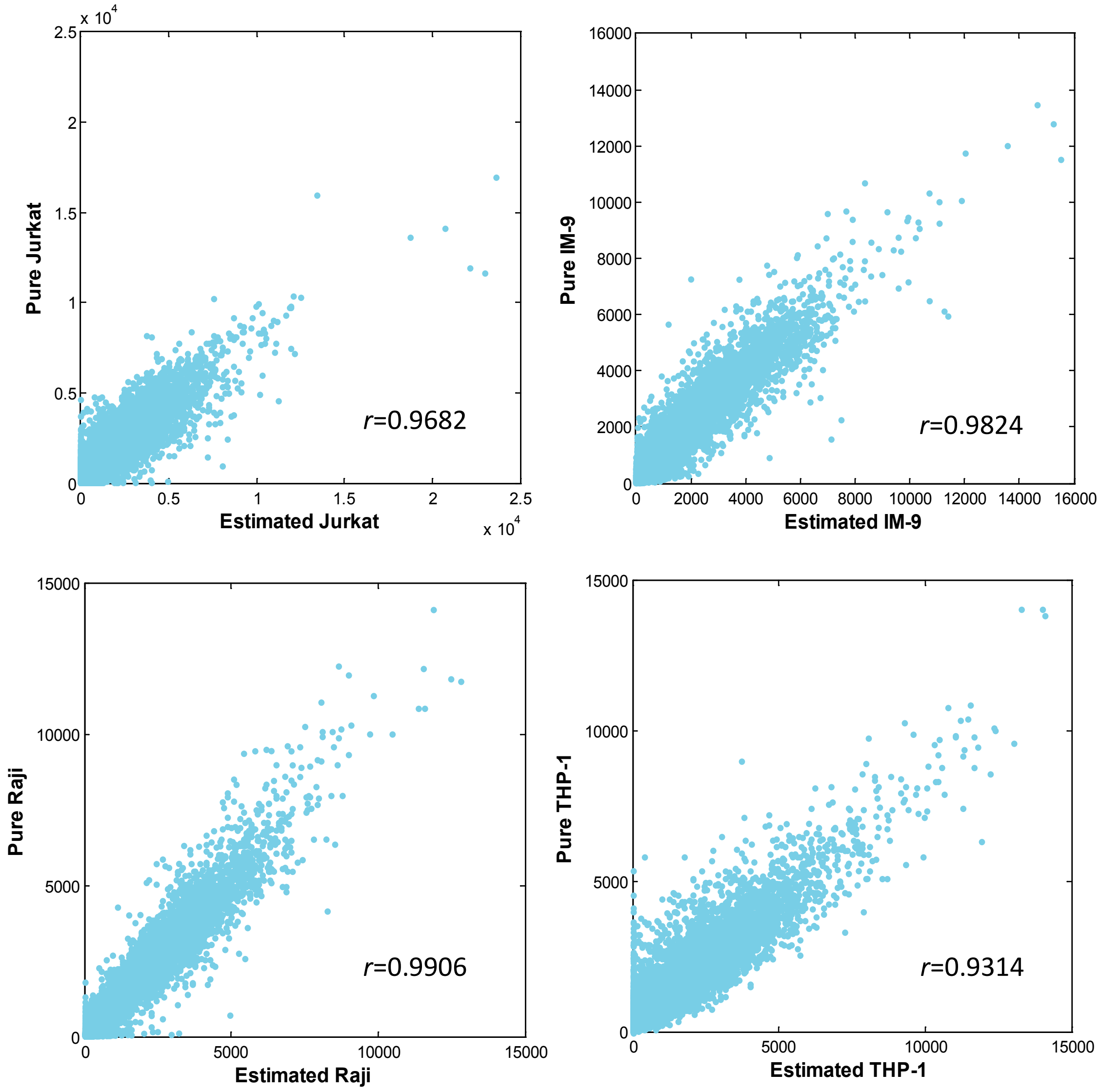

Supplementary Figure 4a.

(a) Raji (B cell)

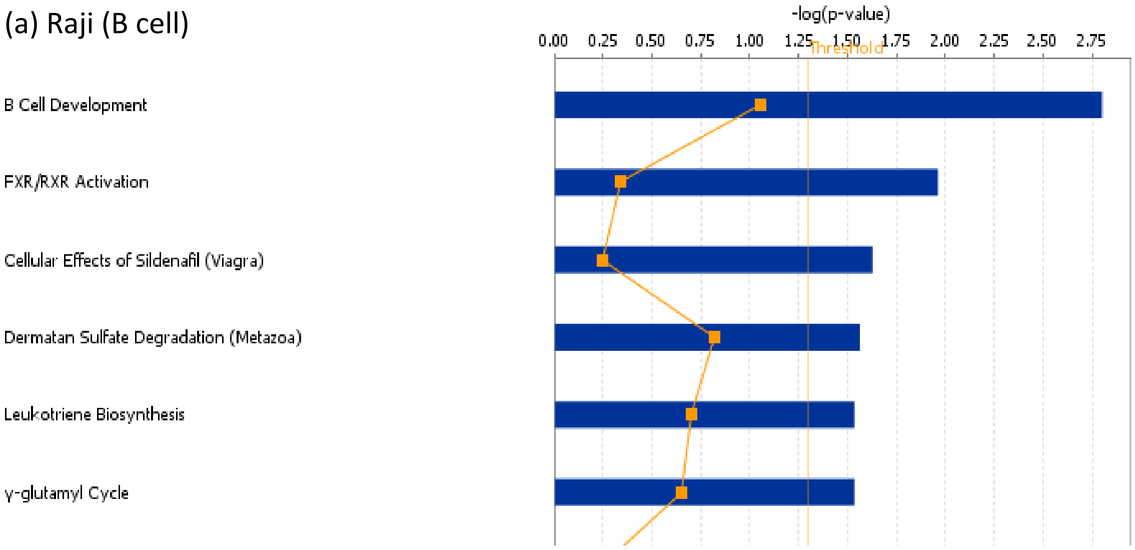


**Top Networks**

| ID | | Associated Network Functions |
|---|---|---|
| 1 | View | Renal and Urological Disease, Metabolic Disease, Neurological Disease |
| 2 | View | Cell-To-Cell Signaling and Interaction, Carbohydrate Metabolism, Lipid Metabolism |
| 3 | View | Cell Death and Survival, Cellular Development, Cell Morphology |
| 4 | View | Cancer, Gastrointestinal Disease |

Supplementary Figure 4b.

(b) Jurkat (T cell)

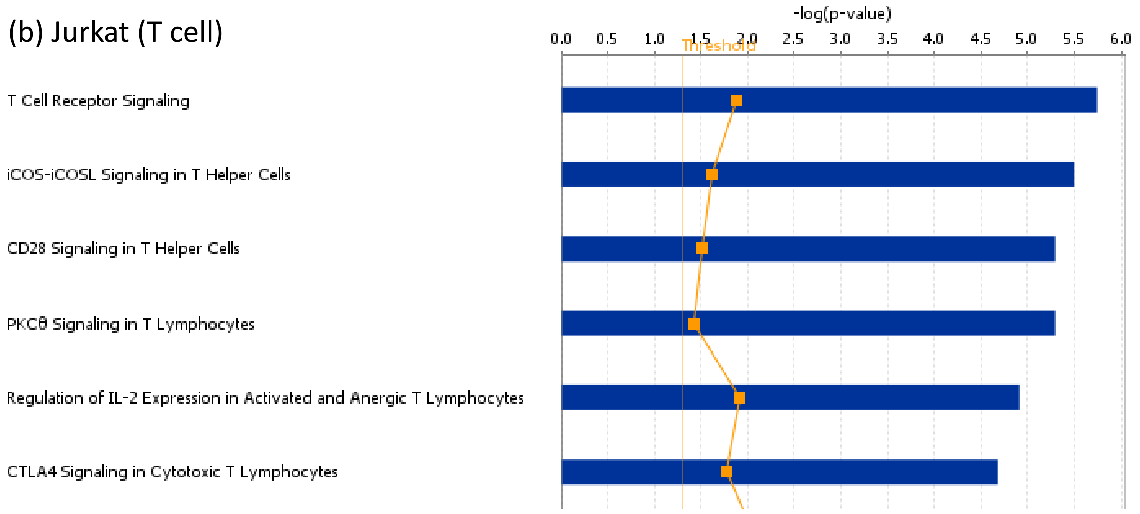


## Top Networks

| ID | | Associated Network Functions |
|---|---|---|
| 1 | View | Cellular Development, Hematological System Development and Function, Hematopoiesis |
| 2 | View | Cellular Growth and Proliferation, Gene Expression, Cellular Development |
| 3 | View | Embryonic Development, Organismal Development, Tissue Development |
| 4 | View | Cancer, Cellular Development, Embryonic Development |
| 5 | View | Energy Production, Lipid Metabolism, Small Molecule Biochemistry |

Supplementary Figure 4c.

(c) THP-1 (monocyte)

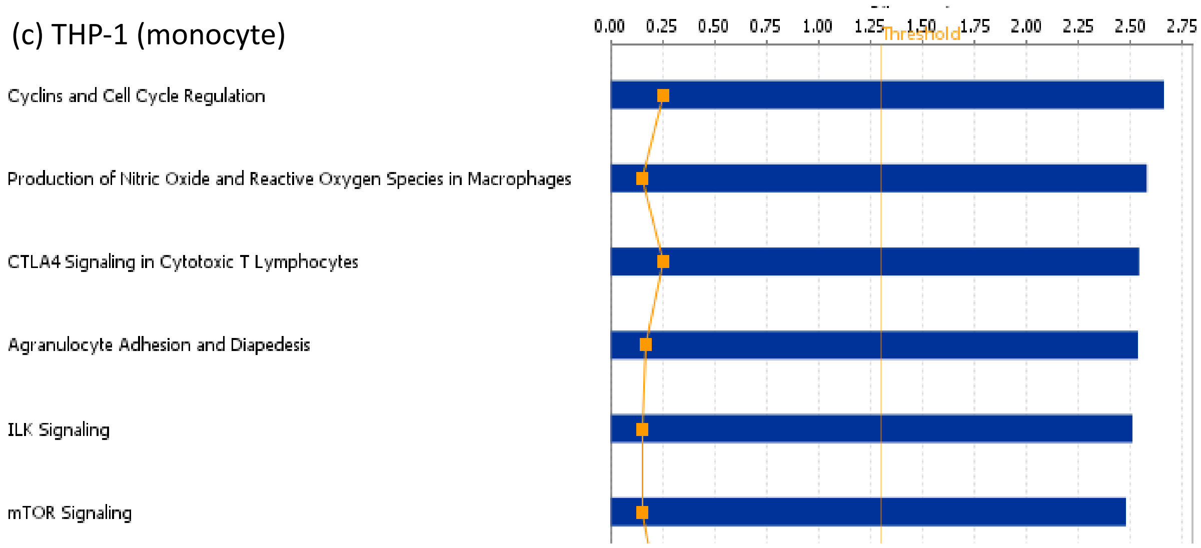


**Top Networks**

| ID | | Associated Network Functions |
|---|---|---|
| 1 | View | Cancer, Cell-To-Cell Signaling and Interaction, Cellular Movement |
| 2 | View | Cancer, Hereditary Disorder, Metabolic Disease |
| 3 | View | Cellular Assembly and Organization, Tissue Development, Lipid Metabolism |
| 4 | View | Cell Cycle, Cell Morphology, Cellular Assembly and Organization |
| 5 | View | Connective Tissue Disorders, Dermatological Diseases and Conditions, Hematological System Development and Function |

Supplementary Figure 4d.

(d) IM-9 (B cell)

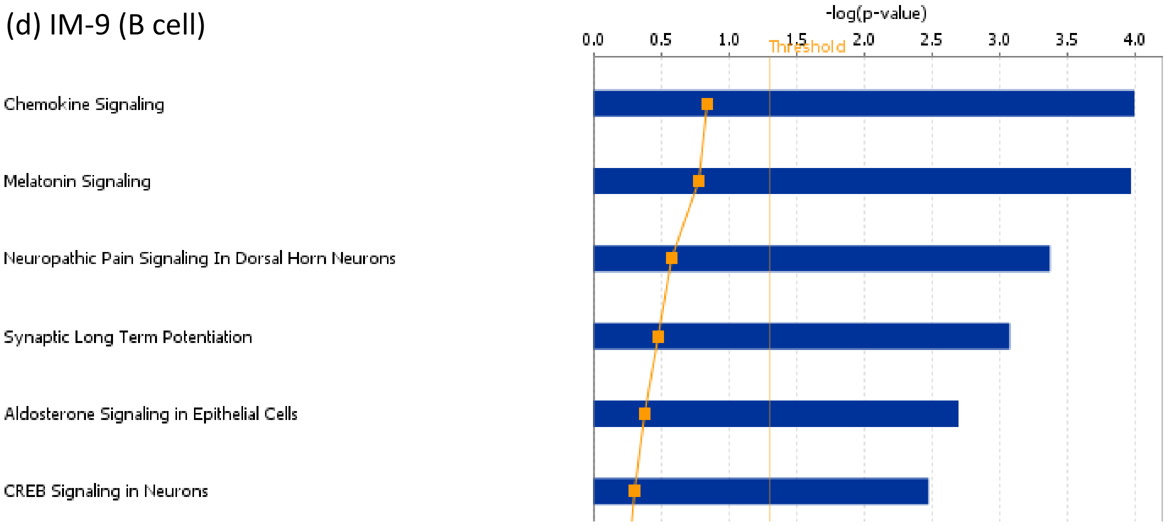


**Top Networks**

| ID | | Associated Network Functions |
|---|---|---|
| 1 | View | Cell-To-Cell Signaling and Interaction, Lipid Metabolism, Molecular Transport |
| 2 | View | Cancer, Dermatological Diseases and Conditions, Cell-To-Cell Signaling and Interaction |
| 3 | View | Cell Cycle, Cell Death and Survival, Lipid Metabolism |
| 4 | View | Cell Cycle, Hereditary Disorder, Neurological Disease |
| 5 | View | Lipid Metabolism, Small Molecule Biochemistry, Endocrine System Development and Function |

Supplementary Figure 5.

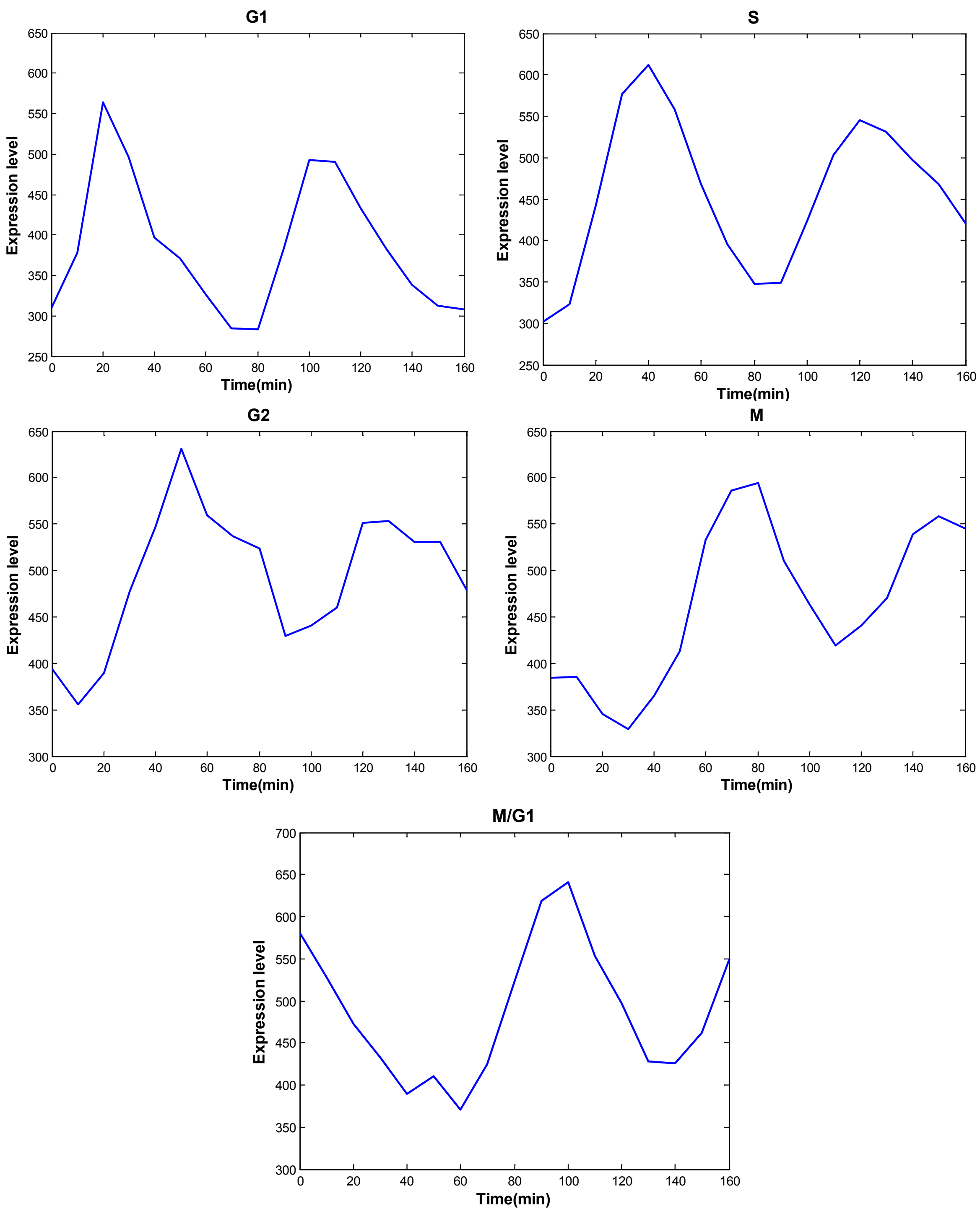

Supplementary Figure 6.

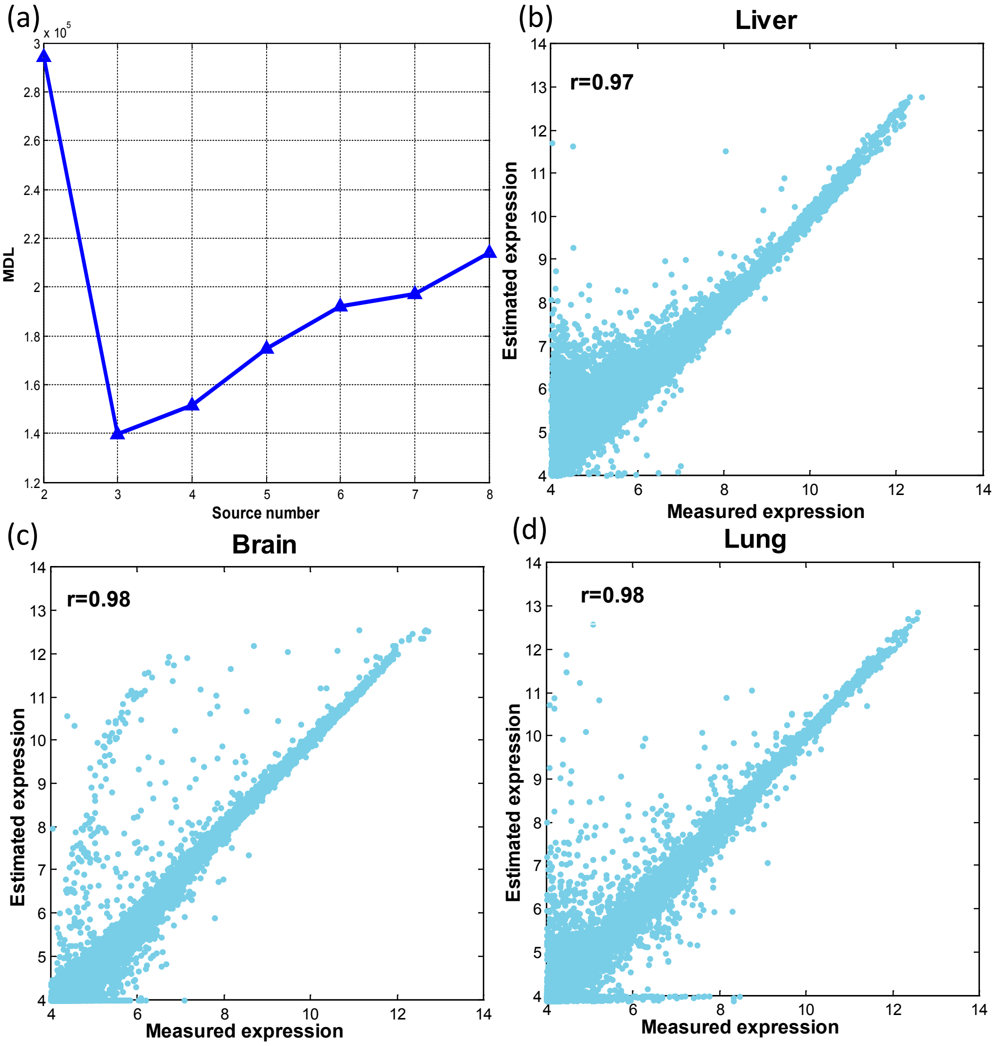

Supplementary Figure 7.

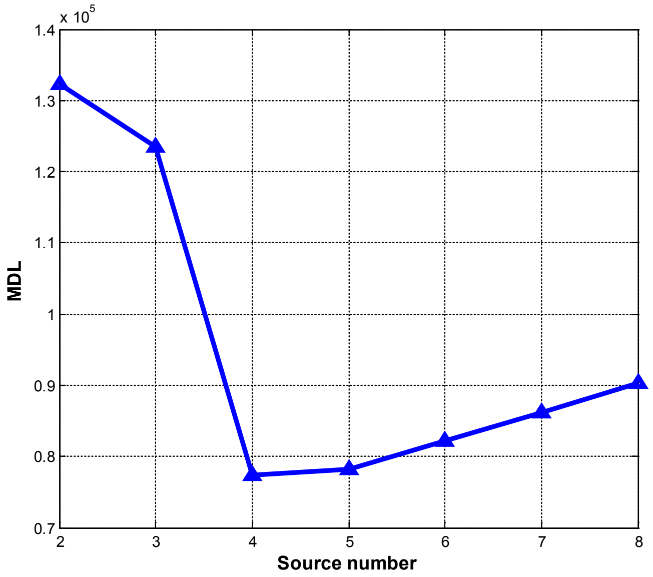


Supplementary Table 1a.

| Proportions (Truth/ estimate) | liver | brain | lung |
|---|---|---|---|
| Mixture 1 | 0.606/0.597 | 0.058/0.063 | 0.336/0.340 |
| Mixture 2 | 0.326/0.325 | 0.626/0.616 | 0.047/0.059 |
| Mixture 3 | 0.027/0.033 | 0.337/0.335 | 0.636/0.632 |

Supplementary Table 1b.

| Proportions (Truth/ estimate) | liver | brain | lung |
|---|---|---|---|
| Mixture 1 | 0.549/0.544 | 0.033/0.038 | 0.419/0.418 |
| Mixture 2 | 0.403/0.403 | 0.581/0.574 | 0.016/0.023 |
| Mixture 3 | 0.017/0.023 | 0.416/0.414 | 0.567/0.563 |

Supplementary Table 1c.

| Proportions (Truth/ estimate) | liver | brain | lung |
|---|---|---|---|
| Mixture 1 | 0.471/0.469 | 0.028/0.034 | 0.501/0.498 |
| Mixture 2 | 0.474/0.472 | 0.518/0.513 | 0.008/0.015 |
| Mixture 3 | 0.036/0.043 | 0.488/0.484 | 0.476/0.473 |

Supplementary Table 2a.

| True/estimated proportions (%) | MixA | MixB | MixC | MixD |
|---|---|---|---|---|
| Jurkat | 25/28.39 | 5/8.40 | 1/2.85 | 0.2/1.73 |
| IM-9 | 12.5/9.62 | 31.7/19.95 | 49.5/45.42 | 33.3/27.99 |
| Raji | 25/24.55 | 47.5/52.22 | 16.5/18.21 | 33.3/33.66 |
| THP-1 | 37.5/37.44 | 15.8/19.43 | 33/33.52 | 33.3/36.62 |

Supplementary Table 2b.

| % | MixA | | | | MixB | | | | MixC | | | | MixD | | | |
|---|---|---|---|---|---|---|---|---|---|---|---|---|---|---|---|---|
| | GT | 1 | 2 | 3 | GT | 4 | 5 | 6 | GT | 7 | 8 | 9 | GT | 10 | 11 | 12 |
| Jurkat | 25 | 30.46 | 29.68 | 30.51 | 5 | 8.09 | 9.23 | 7.92 | 1 | 2.10 | 1.60 | 3.10 | 0.2 | 1.39 | 1.02 | 0.78 |
| IM-9 | 12.5 | 7.12 | 6.46 | 7.99 | 31.7 | 18.51 | 21.72 | 19.63 | 49.5 | 45.43 | 44.02 | 45.73 | 33.3 | 28.18 | 25.02 | 27.21 |
| Raji | 25 | 23.80 | 25.12 | 22.67 | 47.5 | 52.57 | 49.89 | 52.84 | 16.5 | 17.18 | 19.57 | 16.54 | 33.3 | 31.55 | 35.01 | 32.38 |
| THP-1 | 37.5 | 38.62 | 38.73 | 38.83 | 15.8 | 20.83 | 19.17 | 19.60 | 33 | 35.30 | 34.81 | 34.62 | 33.3 | 38.88 | 38.95 | 39.62 |

Supplementary Table 3.

| Subpopulation - specific expression profiles | Estimated vs Pure Jurkat | Estimated vs Pure IM-9 | Estimated vs Pure Raji | Estimated vs Pure THP-1 |
|---|---|---|---|---|
| CAM | 0.97 | 0.98 | 0.99 | 0.93 |

| | | | | |
|---|---|---|---|---|
| Zuckerman et al [1] | 0.96 | 0.96 | 0.95 | 0.93 |

| Mixing proportions | Estimated vs Pure Jurkat | Estimated vs Pure IM-9 | Estimated vs Pure Raji | Estimated vs Pure THP-1 |
|---|---|---|---|---|
| CAM | 1 | 0.97 | 0.99 | 0.99 |
| Zuckerman et al [1] | 0.85 | 0.85 | 0.74 | 0.74 |

Supplementary Table 4a.

| | Jurkat | IM-9 | Raji | THP-1 |
|---|---|---|---|---|
| Jurkat | 1 | 0.9366 | 0.9256 | 0.9160 |
| IM-9 | - | 1 | 0.9588 | 0.9230 |
| Raji | - | - | 1 | 0.9103 |
| THP-1 | - | - | - | 1 |

Supplementary Table 4b.

| | Jurkat | IM-9 | Raji | THP-1 |
|---|---|---|---|---|
| Jurkat | 1 | -0.0840 | -0.0723 | -0.1298 |
| IM-9 | - | 1 | -0.0951 | -0.1955 |
| Raji | - | - | 1 | -0.2527 |
| THP-1 | - | - | - | 1 |

Supplementary Table 4c.

| Estimated vs Pure Jurkat | Estimated vs Pure IM-9 | Estimated vs Pure Raji | Estimated vs Pure THP-1 |
|---|---|---|---|
| 0.9682 | 0.9824 | 0.9906 | 0.9314 |

Supplementary Table 4d.

| Estimated vs Pure Jurkat | Estimated vs Pure IM-9 | Estimated vs Pure Raji | Estimated vs Pure THP-1 |
|---|---|---|---|
| 0.9590 | 0.9841 | 0.9862 | 0.9225 |

Supplementary Table 4e.

| Estimated vs Pure Jurkat | Estimated vs Pure IM-9 | Estimated vs Pure Raji | Estimated vs Pure THP-1 |
|---|---|---|---|
| 0.9521 | 0.9758 | 0.9745 | 0.9633 |

Supplementary Table 4f.

| Estimated vs Pure Jurkat | Estimated vs Pure IM-9 | Estimated vs Pure Raji | Estimated vs Pure THP-1 |
|---|---|---|---|
| 0.9342 | 0.9677 | 0.9403 | 0.9537 |

Supplementary Table 5a.

| Annotation Cluster 1 | Enrichment Score: 1.62 | | Count | P_Value | Benjamini |
|---|---|---|---|---|---|
| UP_SEQ_FEATURE | cross-link:Glycyl lysine isopeptide (Lys-Gly) (interchain with G-Cter in ubiquitin) | RT | 4 | 1.4E-2 | 7.1E-1 |
| SP_PIR_KEYWORDS | isopeptide bond | RT | 4 | 2.4E-2 | 6.6E-1 |
| SP_PIR_KEYWORDS | ubl conjugation | RT | 4 | 4.3E-2 | 7.3E-1 |
| **Annotation Cluster 2** | **Enrichment Score: 1.58** | | **Count** | **P_Value** | **Benjamini** |
| GOTERM_MF_FAT | cyclin-dependent protein kinase regulator activity | RT | 3 | 7.6E-3 | 5.9E-1 |
| GOTERM_MF_FAT | protein kinase regulator activity | RT | 3 | 2.1E-2 | 7.0E-1 |
| GOTERM_MF_FAT | kinase regulator activity | RT | 3 | 2.4E-2 | 6.1E-1 |
| GOTERM_BP_FAT | cell cycle process | RT | 6 | 1.3E-1 | 9.4E-1 |
| **Annotation Cluster 3** | **Enrichment Score: 1.44** | | **Count** | **P_Value** | **Benjamini** |
| GOTERM_BP_FAT | regulation of conjugation | RT | 3 | 1.4E-2 | 9.9E-1 |
| GOTERM_BP_FAT | regulation of conjugation with cellular fusion | RT | 3 | 1.4E-2 | 9.9E-1 |
| GOTERM_BP_FAT | regulation of multi-organism process | RT | 3 | 1.4E-2 | 9.9E-1 |
| GOTERM_BP_FAT | response to pheromone | RT | 3 | 1.0E-1 | 9.5E-1 |
| GOTERM_BP_FAT | response to organic substance | RT | 3 | 2.0E-1 | 9.7E-1 |
| **Annotation Cluster 4** | **Enrichment Score: 1.21** | | **Count** | **P_Value** | **Benjamini** |
| GOTERM_CC_FAT | cellular bud | RT | 8 | 5.4E-5 | 4.6E-3 |
| SP_PIR_KEYWORDS | cell cycle | RT | 7 | 3.9E-3 | 2.9E-1 |
| GOTERM_CC_FAT | site of polarized growth | RT | 6 | 7.7E-3 | 2.8E-1 |
| GOTERM_CC_FAT | cellular bud neck | RT | 5 | 8.3E-3 | 2.1E-1 |
| GOTERM_BP_FAT | interphase of mitotic cell cycle | RT | 4 | 1.6E-2 | 9.2E-1 |
| GOTERM_BP_FAT | interphase | RT | 4 | 1.8E-2 | 8.5E-1 |
| GOTERM_BP_FAT | G1/S transition of mitotic cell cycle | RT | 3 | 2.5E-2 | 8.6E-1 |
| GOTERM_BP_FAT | cell cycle | RT | 8 | 3.4E-2 | 8.9E-1 |
| GOTERM_BP_FAT | regulation of cell cycle | RT | 4 | 6.1E-2 | 9.7E-1 |
| SP_PIR_KEYWORDS | cell division | RT | 4 | 6.9E-2 | 8.0E-1 |
| GOTERM_BP_FAT | mitotic cell cycle | RT | 5 | 7.0E-2 | 9.6E-1 |
| GOTERM_BP_FAT | cell division | RT | 5 | 9.8E-2 | 9.6E-1 |
| GOTERM_BP_FAT | cell cycle process | RT | 6 | 1.3E-1 | 9.4E-1 |

Supplementary Table 5b.

| | Annotation Cluster 1 | Enrichment Score: 5.32 | | | Count | P_Value | Benjamini |
|---|---|---|---|---|---|---|---|
| | GOTERM_BP_FAT | DNA-dependent DNA replication | RT | | 9 | 3.5E-8 | 3.5E-6 |
| | GOTERM_BP_FAT | lagging strand elongation | RT | | 6 | 9.1E-8 | 6.8E-6 |
| | GOTERM_CC_FAT | replication fork | RT | | 7 | 5.5E-7 | 5.2E-5 |
| | KEGG_PATHWAY | DNA replication | RT | | 7 | 7.6E-7 | 9.1E-6 |
| | GOTERM_BP_FAT | DNA strand elongation | RT | | 6 | 1.8E-6 | 6.7E-5 |
| | GOTERM_BP_FAT | DNA strand elongation during DNA replication | RT | | 6 | 1.8E-6 | 6.7E-5 |
| | GOTERM_BP_FAT | base-excision repair | RT | | 3 | 3.7E-3 | 3.6E-2 |
| | KEGG_PATHWAY | Base excision repair | RT | | 3 | 1.8E-2 | 5.3E-2 |
| | Annotation Cluster 2 | Enrichment Score: 4.31 | | | Count | P_Value | Benjamini |
| | GOTERM_BP_FAT | DNA replication | RT | | 14 | 7.8E-13 | 2.3E-10 |
| | GOTERM_BP_FAT | DNA metabolic process | RT | | 17 | 1.2E-9 | 1.8E-7 |
| | GOTERM_BP_FAT | DNA-dependent DNA replication | RT | | 9 | 3.5E-8 | 3.5E-6 |
| | GOTERM_BP_FAT | DNA repair | RT | | 12 | 1.1E-7 | 6.4E-6 |
| | SP_PIR_KEYWORDS | dna replication | RT | | 8 | 1.5E-7 | 1.0E-5 |
| | GOTERM_BP_FAT | response to DNA damage stimulus | RT | | 12 | 5.5E-7 | 2.3E-5 |
| | GOTERM_CC_FAT | replication fork | RT | | 7 | 5.5E-7 | 5.2E-5 |
| | KEGG_PATHWAY | DNA replication | RT | | 7 | 7.6E-7 | 9.1E-6 |
| | GOTERM_CC_FAT | chromosome | RT | | 12 | 5.9E-6 | 2.8E-4 |
| | GOTERM_CC_FAT | chromosomal part | RT | | 11 | 1.3E-5 | 4.2E-4 |
| | GOTERM_MF_FAT | DNA binding | RT | | 13 | 3.2E-5 | 2.8E-3 |
| | GOTERM_CC_FAT | nuclear replication fork | RT | | 5 | 1.2E-4 | 2.8E-3 |
| | SP_PIR_KEYWORDS | nucleus | RT | | 21 | 1.7E-4 | 4.0E-3 |
| | GOTERM_CC_FAT | nuclear chromosome | RT | | 9 | 1.8E-4 | 3.4E-3 |
| | GOTERM_BP_FAT | cellular response to stress | RT | | 12 | 3.0E-4 | 7.6E-3 |
| | GOTERM_CC_FAT | nuclear chromosome part | RT | | 8 | 5.1E-4 | 8.1E-3 |
| | GOTERM_BP_FAT | DNA recombination | RT | | 7 | 6.3E-4 | 9.8E-3 |
| | GOTERM_CC_FAT | intracellular non-membrane-bounded organelle | RT | | 15 | 2.9E-3 | 3.8E-2 |
| | GOTERM_CC_FAT | non-membrane-bounded organelle | RT | | 15 | 2.9E-3 | 3.8E-2 |
| | GOTERM_CC_FAT | replisome | RT | | 3 | 5.2E-3 | 6.0E-2 |

Supplementary Table 5c.

| Annotation Cluster 1 | Enrichment Score: 1.93 | | Count | P_Value | Benjamini |
|---|---|---|---|---|---|
| GOTERM_BP_FAT | cellular amino acid catabolic process | RT | 3 | 8.7E-3 | 4.4E-1 |
| GOTERM_BP_FAT | amine catabolic process | RT | 3 | 1.0E-2 | 4.0E-1 |
| GOTERM_BP_FAT | organic acid catabolic process | RT | 3 | 1.5E-2 | 4.4E-1 |
| GOTERM_BP_FAT | carboxylic acid catabolic process | RT | 3 | 1.5E-2 | 4.4E-1 |
| **Annotation Cluster 2** | **Enrichment Score: 1.02** | | **Count** | **P_Value** | **Benjamini** |
| SP_PIR_KEYWORDS | lyase | RT | 3 | 4.1E-2 | 9.8E-1 |
| SP_PIR_KEYWORDS | amino-acid biosynthesis | RT | 3 | 6.6E-2 | 9.5E-1 |
| GOTERM_BP_FAT | cellular amino acid biosynthetic process | RT | 3 | 7.8E-2 | 9.3E-1 |
| GOTERM_BP_FAT | amine biosynthetic process | RT | 3 | 8.6E-2 | 9.2E-1 |
| GOTERM_BP_FAT | organic acid biosynthetic process | RT | 3 | 1.2E-1 | 9.6E-1 |
| GOTERM_BP_FAT | carboxylic acid biosynthetic process | RT | 3 | 1.2E-1 | 9.6E-1 |
| GOTERM_BP_FAT | nitrogen compound biosynthetic process | RT | 3 | 3.0E-1 | 1.0E0 |
| **Annotation Cluster 3** | **Enrichment Score: 0.87** | | **Count** | **P_Value** | **Benjamini** |
| GOTERM_CC_FAT | integral to membrane | RT | 11 | 3.5E-2 | 8.1E-1 |
| GOTERM_CC_FAT | intrinsic to membrane | RT | 11 | 4.8E-2 | 6.8E-1 |
| UP_SEQ_FEATURE | transmembrane region | RT | 10 | 1.3E-1 | 1.0E0 |
| SP_PIR_KEYWORDS | transmembrane | RT | 10 | 1.3E-1 | 9.9E-1 |
| SP_PIR_KEYWORDS | membrane | RT | 12 | 1.6E-1 | 9.8E-1 |
| SP_PIR_KEYWORDS | transmembrane protein | RT | 9 | 3.7E-1 | 1.0E0 |
| SP_PIR_KEYWORDS | transport | RT | 5 | 4.5E-1 | 1.0E0 |
| **Annotation Cluster 4** | **Enrichment Score: 0.34** | | **Count** | **P_Value** | **Benjamini** |
| SP_PIR_KEYWORDS | mitochondrion | RT | 5 | 3.7E-1 | 1.0E0 |
| UP_SEQ_FEATURE | transit peptide:Mitochondrion | RT | 3 | 4.2E-1 | 1.0E0 |

Supplementary Table 5d.

| Annotation Cluster 1 | Enrichment Score: 1.74 | | Count | P_Value | Benjamini |
|---|---|---|---|---|---|
| GOTERM_BP_FAT | ribosome biogenesis | RT | 20 | 3.0E-4 | 2.0E-1 |
| SP_PIR_KEYWORDS | ribosome biogenesis | RT | 12 | 3.2E-4 | 4.9E-2 |
| GOTERM_BP_FAT | ncRNA processing | RT | 19 | 4.9E-4 | 1.7E-1 |
| GOTERM_BP_FAT | ribonucleoprotein complex biogenesis | RT | 21 | 5.3E-4 | 1.2E-1 |
| GOTERM_BP_FAT | maturation of SSU-rRNA from tricistronic rRNA transcript (SSU-rRNA, 5.8S rRNA, LSU-rRNA) | RT | 9 | 5.4E-4 | 9.5E-2 |
| GOTERM_BP_FAT | maturation of SSU-rRNA | RT | 9 | 6.3E-4 | 9.0E-2 |
| GOTERM_CC_FAT | nucleolus | RT | 16 | 6.8E-4 | 1.3E-1 |
| GOTERM_BP_FAT | rRNA processing | RT | 15 | 9.5E-4 | 1.1E-1 |
| GOTERM_BP_FAT | ncRNA metabolic process | RT | 20 | 1.2E-3 | 1.2E-1 |
| GOTERM_BP_FAT | rRNA metabolic process | RT | 15 | 1.4E-3 | 1.2E-1 |
| GOTERM_BP_FAT | RNA processing | RT | 22 | 5.5E-3 | 3.3E-1 |
| SP_PIR_KEYWORDS | rrna processing | RT | 10 | 6.1E-3 | 2.7E-1 |
| GOTERM_BP_FAT | endonucleolytic cleavage in ITS1 to separate SSU-rRNA from 5.8S rRNA and LSU-rRNA from tricistronic rRNA transcript (SSU-rRNA, 5.8S rRNA, LSU-rRNA) | RT | 5 | 1.0E-2 | 4.3E-1 |
| GOTERM_BP_FAT | endonucleolytic cleavage of tricistronic rRNA transcript (SSU-rRNA, 5.8S rRNA, LSU-rRNA) | RT | 5 | 1.2E-2 | 4.6E-1 |
| GOTERM_BP_FAT | endonucleolytic cleavages during rRNA processing | RT | 5 | 1.2E-2 | 4.6E-1 |
| GOTERM_CC_FAT | small-subunit processome | RT | 5 | 1.6E-2 | 6.6E-1 |
| GOTERM_CC_FAT | preribosome | RT | 8 | 2.2E-2 | 6.6E-1 |
| GOTERM_CC_FAT | nuclear lumen | RT | 20 | 2.6E-2 | 6.5E-1 |
| GOTERM_CC_FAT | 90S preribosome | RT | 6 | 2.9E-2 | 6.2E-1 |
| GOTERM_BP_FAT | cleavages during rRNA processing | RT | 5 | 4.5E-2 | 8.3E-1 |

Supplementary Table 6.

| GSE19830 | Liver | Brain | Lung |
|---|---|---|---|
| Pure Sample 1 | 1 | 0 | 0 |
| Pure Sample 2 | 0 | 1 | 0 |
| Pure Sample 3 | 0 | 0 | 1 |
| **True/estimate** | | | |
| Mixture 1 | 0.05/0.0545 | 0.25/0.2881 | 0.7/0.6574 |
| Mixture 2 | 0.7/0.6938 | 0.05/0.0318 | 0.25/0.2744 |
| Mixture 3 | 0.25/0.1966 | 0.7/0.7527 | 0.05/0.0507 |
| Mixture 4 | 0.7/0.6163 | 0.25/0.3277 | 0.05/0.0560 |
| Mixture 5 | 0.45/0.4054 | 0.45/0.5054 | 0.1/0.0893 |
| Mixture 6 | 0.55/0.5369 | 0.2/0.2272 | 0.25/0.2359 |
| Mixture 7 | 0.5/0.4723 | 0.3/0.3414 | 0.2/0.1863 |
| Mixture 8 | 0.55/0.5064 | 0.3/0.3513 | 0.15/0.1423 |
| Mixture 9 | 0.5/0.4495 | 0.4/0.4620 | 0.1/0.0885 |
| Mixture 10 | 0.6/0.5295 | 0.35/0.4278 | 0.05/0.0427 |
| Mixture 11 | 0.65/0.5700 | 0.34/0.4234 | 0.01/0.0066 |

Supplementary Table 7.

| Cell type | Marker gene symbol (Rattus norvegicus) | Probe set (Affymetrix Rat 230 2.0) |
|---|---|---|
| Neuron | Nefl | 1370058_at<br>1370059_at |
| | Snap25 | 1387073_at |
| | Nefm | 1367845_at |
| Astrocyte | Aqp4 | 1372190_at |
| | S100b | 1386903_at |
| | Id4 | 1375120_at<br>1375183_at<br>1385923_at |
| Oligodendrocyte | Mog | 1398257_at |
| | Mag | 1368861_a_at |
| | Mobp | 1368263_a_at<br>1370434_a_at<br>1370500_a_at |
| Microglia | Cd37 | 1368555_at |
| | Itgam | 1388046_at |
| | Cd53 | 1368518_at |

## Supplementary Figure legends

**Supplementary Figure 1**: Unreliability of the supervised method on GSE19830 in the presence of uncertainties in measured subpopulation proportions [2]. The constituent proportions were randomly perturbed with ±0.05~0.3 or one of the ten mixtures was replaced with arbitrary constituent proportions. If the proportion is less than 0, it is set to 0. After perturbation, each row sum of the mixing matrix was normalized to 1. (a-c) The estimated liver/brain/lung-specific expression profiles were compared with the ground truth using correlation coefficients estimated over all genes, where the constituent proportions were randomly perturbed with ±0.05~0.3. The experiment was repeated 100 times to reflect true performance with each perturbation rate. (d-f) The estimated liver/brain/lung-specific expression profiles are compared with ground truth using correlation coefficient over marker genes, where the constituent proportions are randomly perturbed with ±0.05~0.3; and to reflect the true performance, under each perturbation rate, the experiment is repeated 100 times. (g-i) Boxplot of the correlation coefficients between the estimated liver/brain/lung-specific expression profiles and ground truth over all genes, where one of the ten mixtures is replaced with arbitrary constituent proportions and the experiment is repeated 500 times. (j-l) The Boxplots of correlation coefficients between the estimated liver/brain/lung-specific expression profiles and ground truth over marker genes, where one of the ten mixtures is replaced with arbitrary constituent proportions and the experiment is repeated 500 times.

**Supplementary Figure 2**: Unreliability of supervised method on GSE19380 in the presence of uncertainties in selected *a priori* marker genes/signatures [3]. The proportion estimates using the marker genes are compared with the ground truth using correlation coefficient anlaysis. (a) The reference signatures (expression levels of marker genes) are perturbed with ±1~50% variations, (b) Two of the marker genes are replaced with arbitrary genes. The experiment was repeated 1000 times to generate a histogram of the correlation coefficients between the proportion estimates and ground truth.

**Supplementary Figure 3**: CAM validation on GSE11058 [4] – subpopulation-specific profiles. Scatter plots of the measured (by cell sorting) and estimated (by CAM) subpopulation-specific gene expression profiles for the four source subpopulations.

**Supplementary Figure 4**: Ingenuity Pathway Analysis (IPA) on CAM detected marker genes on GSE11058. (a) Top pathways and networks enriched by the CAM-detected marker genes associated with Raji (B cell). (b) Top pathways and networks enriched by the CAM detected marker genes associated with Jurkat (T cell). (c) Top pathways and networks enriched by the CAM detected marker genes associated with THP-1 (monocyte). (d) Top pathways and networks enriched by the CAM detected marker genes associated with IM-9 (B cell).

**Supplementary Figure 5**: CAM validation on cell cycle data – comparison with peer reports. The phase-specific time-course patterns generated by the marker genes selected by Spellman *et al.* [5].

**Supplementary Figure 6**: CAM validation on GSE19830 [2] - MDL curve for detecting the number of sources (*e.g.*, subpopulations) and blindly uncovered subpopulation-specific expression profiles. The mixtures were obtained from biologically mixed expression profiles of the three (3) subpopulations, *i.e.*, liver, brain and lung tissues, with each source tissue sample having three replicates. (a) The MDL values are calculated over $K = 2, 3, \ldots, 8$, with minimum

value corresponding to $K = 3$. (b-d) Scatter plots of the measured (by tissue extraction) and estimated (by *in silico* CAM) subpopulation-specific gene expression profiles for the three source subpopulations, the same results as Figure 2d but with log-transformation [2].

**Supplementary Figure 7**: CAM validation on cell cycle data - MDL curve for detecting the number of phases in yeast cell cycles.

## Supplementary Table legends

**Supplementary Table 1**: CAM validation on synthetic GSE19830 [2]. (a) Validation results on $30^0$ rotation: proportion estimates versus ground truth. (b) Validation results on $45^0$ rotation: proportion estimates versus ground truth. (c) Validation results on $60^0$ rotation: proportion estimates versus ground truth.

**Supplementary Table 2**: CAM validation on GSE11058 – proportion estimates. (a) The true and estimated proportions of four cell lines in the 4 mixture samples (average profile of 3 replicates). (b) The sample-specific true and estimated proportions of four cell lines in the original 12 mixture samples.

**Supplementary Table 3**: CAM validation on GSE11058 – comparison with peer method [1]. Despite being completely unsupervised method, CAM even slightly outperformed the supervised method that requires known reference marker gene signatures to operate.

**Supplementary Table 4**: Correct assessment using marker genes versus misleading assessment when using all genes – on GSE11058. (a) The correlation coefficients between the expression profiles of different pure cell lines, when assessed using all genes (including many invariantly expressed or house-keeping genes), are significantly high - this is clearly misleading. (b) The correlation coefficients between the expression profiles of different pure cell lines, when assessed using only marker genes, are significantly low - this correctly reflects the signature differences among different cell lines. (c) The high correlation coefficients between the measured and estimated (by CAM) expression profiles associated with the four pure cell lines, when assessed over all genes, are as expected. (d) The high correlation coefficients between the measured and estimated (by supervised method) expression profiles associated with the four pure cell lines, when assessed over all genes, are expected. (e) The retained high correlation coefficients between the measured and estimated (by CAM) expression profiles associated with the four pure cell lines, when assessed over only true marker genes, reflect truly CAM's ability to blindly detect marker genes and accurately deconvolute mixed expression profiles into subpopulation-specific expression profiles. (f) The retained high correlation coefficients between the measured and estimated (by supervised method) expression profiles associated with the four pure cell lines, when assessed using only true marker genes, are expected.

**Supplementary Table 5**: CAM Marker Gene Enrichment Analysis on cell cycle data by DAVID. 187 phase-specific markers genes are blindly detected by CAM, and the functional enrichment analysis on these marker genes is done using the DAVID knowledge base. (a) Enriched functions associated with G1 phase. (b) Enriched functions associated with S phase. (c) Enriched functions associated with G2/M phase. (d) Enriched functions associated with M/G1 phase.

**Supplementary Table 6**: CAM validation on original dataset GSE19830 [2]. The mixtures were obtained from biologically mixed expression profiles.

**Supplementary Table 7**: *A priori* marker gene list used by Kuhn et al. [3] on GSE19380.

## References


1 Zuckerman, N. S., Noam, Y., Goldsmith, A. J. & Lee, P. P. A self-directed method for cell-type identification and separation of gene expression microarrays. *PLoS Comput Biol* **9**, e1003189, doi:10.1371/journal.pcbi.1003189 (2013).

2 Shen-Orr, S. S. *et al.* Cell type-specific gene expression differences in complex tissues. *Nat Methods* **7**, 287-289, doi:10.1038/nmeth.1439 (2010).

3 Kuhn, A., Thu, D., Waldvogel, H. J., Faull, R. L. & Luthi-Carter, R. Population-specific expression analysis (PSEA) reveals molecular changes in diseased brain. *Nat Methods* **8**, 945-947, doi:nmeth.1710 (2011).

4 Abbas, A. R., Wolslegel, K., Seshasayee, D., Modrusan, Z. & Clark, H. F. Deconvolution of blood microarray data identifies cellular activation patterns in systemic lupus erythematosus. *PLoS One* **4**, e6098, doi:10.1371/journal.pone.0006098 (2009).

5 Spellman, P. T. *et al.* Comprehensive identification of cell cycle-regulated genes of the yeast Saccharomyces cerevisiae by microarray hybridization. *Mol Biol Cell* **9**, 3273-3297 (1998).

# Supplementary Method

## Mathematical modelling of transcriptional heterogeneity identifies novel markers and subpopulations in complex tissues

Niya Wang, Eric P. Hoffman, Lulu Chen, Li Chen, Zhen Zhang, Chunyu Liu, Guoqiang Yu, David M. Herrington, Robert Clark, and Yue Wang

**Formal proofs of CAM theorems.**

**Lemma 1 (Scatter compression and rotation).** *Suppose that pure subpopulation expressions are non-negative, and* $\boldsymbol{x}(i) = \boldsymbol{a}_1 s_1(i) + \ldots + \boldsymbol{a}_k s_k(i) + \ldots + \boldsymbol{a}_K s_K(i)$ *where* $\boldsymbol{a}_k$*'s are linearly independent and non-negative, then, the scatter simplex of pure subpopulation expressions is compressed and rotated to form the scatter simplex of mixed expressions whose vertices coincide with* $\boldsymbol{a}_k$*'s.*

Proof of Lemma 1. By the definition of a convex set (i.e., simplex) [1-3], and since $s_k(i) \geq 0$, $s_1(i) + \ldots + s_k(i) + \ldots + s_K(i) = 1$, and $\boldsymbol{x}(i) = \boldsymbol{a}_1 s_1(i) + \ldots + \boldsymbol{a}_k s_k(i) + \ldots + \boldsymbol{a}_K s_K(i)$, then every $\boldsymbol{x}(i)$ belongs to the simplex $\mathcal{H}(\mathcal{A}_K)$ defined by $\boldsymbol{a}_k$*'s* where

$$\mathcal{H}\{\mathcal{A}_K\} = \left\{\sum_{k=1}^{K} \alpha_k \boldsymbol{a}_k \mid \boldsymbol{a}_k \in \mathcal{A}_K,\ \alpha_k \geq 0,\ \sum_{k=1}^{K} \alpha_k = 1\right\}. \quad \text{(S1)}$$

Since $\boldsymbol{a}_1, \ldots, \boldsymbol{a}_K$ are linearly independent, it follows that

$$\sum_{k=1}^{K} \alpha_k \boldsymbol{a}_k = \boldsymbol{0} \text{ iff } \alpha_k = 0\ \forall k \quad \text{(S2)}$$

that in turn implies that $\forall k$

$$\boldsymbol{a}_k = \sum_{k=1}^{K} \alpha_k' \boldsymbol{a}_k \text{ iff } [\alpha_1', \ldots, \alpha_K']^T = \boldsymbol{e}_k,\ \forall k, \quad \text{(S3)}$$

*i.e.*, $\boldsymbol{a}_k$ can only be a trivial convex combination of $\boldsymbol{a}_k$*'s*. Again, by the definition of a convex set, $\boldsymbol{a}_k$*'s* are therefore the vertices of the convex set $\mathcal{H}(\mathcal{A}_K)$.

**Theorem 1 (Unsupervised identifiability).** *Suppose that pure subpopulation expressions are non-negative and subpopulation-specific marker genes exist for each constituting subpopulation, and that* $\boldsymbol{x}(i) = \boldsymbol{a}_1 s_1(i) + \ldots + \boldsymbol{a}_k s_k(i) + \ldots + \boldsymbol{a}_K s_K(i)$ *where* $\boldsymbol{a}_k$*'s are linearly independent and non-negative. Then, the vertices of the scatter simplex of mixed expressions host subpopulation-specific marker genes and coincide with the* $\boldsymbol{a}_k$*'s. the* $\boldsymbol{a}_k$*'s can be readily estimated from marker gene expression values with appropriate rescaling.*

Proof of Theorem 1. Since $\exists i_{\text{MG-}k}$, $\boldsymbol{s}(i_{\text{MG-}k}) = \boldsymbol{e}_k$, $\forall k$, and $\boldsymbol{x}(i) = \boldsymbol{a}_1 s_1(i) + \ldots + \boldsymbol{a}_k s_k(i) + \ldots + \boldsymbol{a}_K s_K(i)$, we have

$$\boldsymbol{x}(i_{\text{MG-}k}) = \boldsymbol{a}_k . \quad \text{(S4)}$$

Then, for any $z \in \mathcal{H}\{\mathcal{A}_K\} = \left\{\sum_{k=1}^{K} \alpha_k \boldsymbol{a}_k \mid \boldsymbol{a}_k \in \mathcal{A}_K,\ \alpha_k \geq 0,\ \sum_{k=1}^{K} \alpha_k = 1\right\}$, we have

$$\begin{aligned} z &= \sum_{k=1}^{K} \alpha_k \boldsymbol{a}_k \\ &= \sum_{k=1}^{K} \alpha_k \boldsymbol{x}(i_{\text{MG-}k}) \\ &= \sum_{i=1}^{N} \alpha_i' \boldsymbol{x}(i), \text{ where } \alpha_i' = \begin{cases} \alpha_k, & i \in \{i_{\text{MG-}k}\}, \\ 0, & i \notin \{i_{\text{MG-}k}\}, \end{cases} \end{aligned} \tag{S5}$$

that implies $z \in \mathcal{H}\{\mathcal{X}_N\} = \left\{\sum_{i=1}^{N} \alpha_i' \boldsymbol{x}(i) \mid \boldsymbol{x}(i) \in \mathcal{X}_N,\ \alpha_i' \geq 0,\ \sum_{i=1}^{N} \alpha_i' = 1\right\}$, *i.e.*, $\mathcal{H}\{\mathcal{A}_K\} \subseteq \mathcal{H}\{\mathcal{X}_N\}$.

Conversely, for any $z \in \mathcal{H}\{\mathcal{X}_N\} = \left\{\sum_{i=1}^{N} \alpha_i\, \boldsymbol{x}(i) \mid \boldsymbol{x}(i) \in \mathcal{X}_N,\ \alpha_i \geq 0,\ \sum_{i=1}^{N} \alpha_i = 1\right\}$, we have

$$\begin{aligned} z &= \sum_{i=1}^{N} \alpha_i\, \boldsymbol{x}(i) \\ &= \sum_{i=1}^{N} \alpha_i \sum_{k=1}^{K} s_k(i) \boldsymbol{a}_k \\ &= \sum_{k=1}^{K} \left[\sum_{i=1}^{N} \alpha_i s_k(i)\right] \boldsymbol{a}_k \\ &= \sum_{k=1}^{K} \beta_k \boldsymbol{a}_k, \text{ where } \beta_k = \sum_{i=1}^{N} \alpha_i s_k(i) \text{ and } \sum_{k=1}^{K} \beta_k = 1, \end{aligned} \tag{S6}$$

that then implies $z \in \mathcal{H}\{\mathcal{A}_K\} = \left\{\sum_{k=1}^{K} \alpha_k \boldsymbol{a}_k \mid \boldsymbol{a}_k \in \mathcal{A}_K,\ \alpha_k \geq 0,\ \sum_{k=1}^{K} \alpha_k = 1\right\}$, *i.e.*, $\mathcal{H}\{\mathcal{X}_N\} \subseteq \mathcal{H}\{\mathcal{A}_K\}$. Combining $\mathcal{H}\{\mathcal{A}_K\} \subseteq \mathcal{H}\{\mathcal{X}_N\}$ and $\mathcal{H}\{\mathcal{X}_N\} \subseteq \mathcal{H}\{\mathcal{A}_K\}$ gives $\mathcal{H}\{\mathcal{X}_N\} = \mathcal{H}\{\mathcal{A}_K\}$. Together with Lemma 1, this readily completes the proof of Theorem 1.

**Data preprocessing.** To obtain a reliable set of marker gene indices, some pre-processing steps are required, including normalization and removal of minimally-expressed and outlier genes. Normalizing gene expression data by global mean/mode is one of the simplest normalization methods [4]. It is performed based on the histogram mean/mode of the raw measured gene expression profile associated with a sample [4-6] so that all the samples have the same mean/mode in signal intensity. Other normalization methods, such as PLIER, RMA and MAS5 on Affymetrix, or lumi Bioconductor package on Illumina can also be used to process the raw expression data. If log-transformed signals were used in normalization, they should be converted back to the raw measured values via anti-log transformation [7-9]. Only raw measured expression values can be used under a linear/additive latent variable model [7-9] and our CAM approach adopts the same strategy.

When the number of heterogeneous samples is large, dimension reduction method is required to reduce the sample dimension. In CAM package, we apply principal component analysis (PCA) to computing eigenvalues and eigenvectors of mixed expression matrix **X**. Users can also choose other methods to reduce sample dimension before performing CAM.

One simple approach is to randomly select $K$+ samples for CAM, although this method cannot take advantage of all information from the complete sample set. Alternatively, one can group all samples into sample clusters, and use the sample cluster centers as pseudo samples to perform CAM. Other strategies, such as Nonnegative matrix factorization (NMF) or random projection can also be used to reduce the sample dimension.

**Aggregation of gene expression vectors.** Let the "connection" $c(i,m)$ indicate how well the $m$th gene vector is suited to be the exemplar for the $i$th gene vector; the "responsibility" $r(i,m)$ reflects the accumulated evidence for how well-suited the $m$th gene vector is to serve as the exemplar for the $i$th gene vector; the "handiness" $h(i,m)$ reflects the accumulated evidence for how appropriate the $i$th gene vector chooses $m$th gene vector as its exemplar. Then, the responsibilities $r(i,m)$ are computed based on

$$r(i,m) \leftarrow c(i,m) - \max_{m' \neq m} \{h(i,m') + c(i,m')\}, \tag{S7}$$

where the handiness $a(i,m)$ is initialized to zero and the competitive update rule (equation (S7)) is purely data-driven. While the responsibility update (equation (S7)) allows all candidate exemplars to compete for ownership of a gene vector, the handiness update rule

$$h(i,m) \leftarrow \min\left\{0, r(m,m) + \sum_{i' \notin \{i,m\}} \max\{0, r(i',m)\}\right\} \tag{S8}$$

collects evidence from gene vectors to support a good exemplar, where the "self-handiness" is updated differently $h(m,m) \leftarrow \sum_{i' \notin m} \max\{0, r(i',m)\}$. Then, the handiness and responsibility are combined to identify examplars $m^* = \arg\max_m \{h(i,m) + r(i,m)\}$. The update rules are repeated iteratively and terminated when no change occurs for about 10 iterations [10,11].

**Detecting the number of subpopulations by MDL.** MDL calculates the total number of 'bits' that are required to encode/explain both the 'data' and 'model' [12,13]. When the model is given (or estimated), only the information about 'mismatch' between model and data needs to be explained (or encoded). The first term (negative joint likelihood) in the MDL determines exactly the 'bits' needed to explain the 'data' conditioned on the given model. The second and third terms represent the 'penalty' on the model complexity, *i.e.*, the total number of bits used to explain the model. Each of these terms involves two multiplicative factors: the number of free-adjustable parameters and the original data points used to estimate the parameters (or the original data points the parameters can and are used to 'explain').

$$\mathrm{MDL}(K) = -\log\left(\mathcal{L}\left(\boldsymbol{X}_M \,|\, \boldsymbol{\theta}(K)\right)\right) + \frac{(K-1)J}{2}\log(M) + \frac{KM}{2}\log(J) \qquad \text{(S9)}$$

Specifically, when estimating the mixing matrix (*i.e.*, the column vectors $\boldsymbol{a}_k$*'s*) that is parameterized by ($K$-1)$J$ independent entries, we use some form of vector-average operation (*i.e.*, $\min_{\alpha_1,\ldots\alpha_K} \left\| \boldsymbol{g}_m - \sum_{k=1}^{K} \alpha_k \boldsymbol{g}_k \right\|_2$ ), where the scalar entry $a_k(j)$ is estimated from $M$ scalar entries $g_m(j)$ for a given $j$, contributing total ($K$-1)$J$log($M$)/2 bits. Similarly, when estimating the subpopulation-specific gene expression profiles (the row vectors $\boldsymbol{s}_k$) with total $KM$ entries, we use a vector-average operation (*i.e.*, solving linear equations), where the scalar entry $s_k(m)$ is estimated involving only $J$ scalar entries $g_m(j)$ for a given $m$, contributing total $KM$log($J$)/2 bits.

**Performance assessment.** $E$1 is a measurement of the similarity between the estimated mixing matrix and the true mixing matrix (Method). This measurement is invariant to permutation and scaling, thus reflecting how close the unmixing matrix W is to the inverse of the true mixing matrix $A^{-1}$. It is nonnegative and achieves value 0 when W and $A^{-1}$ are identical, with an upper bound of 2$N$($N$-1) where $N$ is the data or source dimension.

**Instability of the leading peer <u>supervised</u> method.** In the report on supervised method relying on *a priori* marker gene signatures – GSE19380 [8], while the estimated proportions in samples 17, 19, 21, 23 are close to ground truth, the estimated proportions in samples 18, 20, 22, 24 are significantly from the ground truth. Hence, the indices and expression levels of the marker genes may be either condition dependent and/or sensitive to poor sample quality. To further test whether *a priori* markers are sensitive to potential nonlinearity (*e.g.*, signal saturation) of biological mixing process (while a linear model is assumed and widely adopted), we overlaid the marker genes used by Kuhn *et al.* onto both the scatter plot of expression profiles derived from source tissues and the scatter plot of expression profiles derived from mixed tissues. We found that while the marker genes used by Kuhn *et al.* are located at the vertices of the scatter plot of pure tissue gene expression data, they clearly deviate from the vertices of the scatter plot of mixed tissue expressions.

**CAM analysis on GSE19380.** Rat primary neurons, astrocytes, oligodendrocytes and microglia were cultured separately and their RNAs are extracted to generate expression profiles for the four subpopulations using Affymetrix Rat 230 2.0 microarrays. These reference RNAs

were then mixed to generate composite samples. This gene expression dataset was comprised of 26 profiles: 4 replicates for each of the 4 subpopulations and 10 mixed RNA preparations. However, sample 8 is claimed to be of poor quality by the authors of Kuhn *et al.* [8], and accordingly samples 18, 20, 22 and 24, as well as microglia subpopulations, were removed from further analysis.

**CAM analysis on GSE11058.** Adopting the same procedure as in Abbas et al. [14], we use MAS 5.0 to perform data normalization without logarithm transformation of the data, ensuring a valid linear latent variable model [9]. As aforementioned, probe sets with small norms are easy to be contaminated by noise and those with very large norms may be outliers. Thus, in our analysis, we remove the probe sets with small norms and very large norms, and leaving approximately 13,000 probe sets for further analysis.

To test whether the pure subpopulation expression profiles are dependent, we calculate the cross-correlation coefficients between different sources. We can see from Supplementary Table S4a that even among different pure subpopulations the expression values are highly correlated when assessed using all genes. This should be expected because most genes are not differentially expressed. In contrast, when assessed using only the marker genes reported in Supplementary Table S4b, the cross-correlation is very low, reflecting the 'independency' among different subpopulations.

To assess whether the performance of our unsupervised method CAM is comparable to that achieved by the supervised methods, we compare the results obtained using CAM (without using any form of *a priori* information, Supplementary Fig. S3) and the results obtained using correct supervised information. From Supplementary Tables S4e and S4f, we can see that CAM not only performs comparably with supervised method and can modestly outperforms these methods.

## References


1 Chan, T.-H., Ma, W.-K., Chi, C.-Y. & Wang, Y. A Convex Analysis Framework for Blind Separation of Non-Negative Sources. *IEEE Trans Signal Processing* **56**, 5120-5134 (2008).

2 Boyd, S. & Vandenberghe, L. *Convex Optimization*. 1st edn, (Cambridge University Press, 2004).

3 Wang, F. Y., Chi, C. Y., Chan, T. H. & Wang, Y. Nonnegative least-correlated component analysis for separation of dependent sources by volume maximization. *IEEE Trans Pattern Anal Mach Intell* **32**, 875-888, doi:10.1109/TPAMI.2009.72 (2010).

4 Quackenbush, J. Microarray data normalization and transformation. *Nat Genet* **32 Suppl**, 496-501, doi:10.1038/ng1032 (2002).
5 Wang, Y., Lu, J., Lee, R., Gu, Z. & Clarke, R. Iterative normalization of cDNA microarray data. *IEEE Trans Inf Technol Biomed* **6**, 29-37 (2002).
6 Park, T. *et al.* Evaluation of normalization methods for microarray data. *BMC Bioinformatics* **4**, 33, doi:10.1186/1471-2105-4-33 (2003).
7 Ahn, J. *et al.* DeMix: deconvolution for mixed cancer transcriptomes using raw measured data. *Bioinformatics* **29**, 1865-1871, doi:10.1093/bioinformatics/btt301 (2013).
8 Kuhn, A., Thu, D., Waldvogel, H. J., Faull, R. L. & Luthi-Carter, R. Population-specific expression analysis (PSEA) reveals molecular changes in diseased brain. *Nat Methods* **8**, 945-947, doi:nmeth.1710 (2011).
9 Zhong, Y. & Liu, Z. Gene expression deconvolution in linear space. *Nat Methods* **9**, 8-9; author reply 9, doi:10.1038/nmeth.1830 (2012).
10 Chen, L. *et al.* CAM-CM: a signal deconvolution tool for in vivo dynamic contrast-enhanced imaging of complex tissues. *Bioinformatics* **27**, 2607-2609, doi:btr436 (2011).
11 Wang, N. *et al.* The CAM software for nonnegative blind source separation in R-Java. *J. Machine Learning Research* **14**, 2899-2903 (2013).
12 Wax, M. & Kailath, T. Detection of signals by information theoretic criteria. *IEEE Trans Acoustics, Speech, and Signal Processing* **33**, 387-392 (1985).
13 Chen, L. *et al.* Tissue-specific compartmental analysis for dynamic contrast-enhanced MR imaging of complex tumors. *IEEE Trans Med Imaging* **30**, 2044-2058, doi:10.1109/TMI.2011.2160276 (2011).
14 Abbas, A. R., Wolslegel, K., Seshasayee, D., Modrusan, Z. & Clark, H. F. Deconvolution of blood microarray data identifies cellular activation patterns in systemic lupus erythematosus. *PLoS One* **4**, e6098, doi:10.1371/journal.pone.0006098 (2009).

# Supplementary Discussion

## Mathematical modelling of transcriptional heterogeneity identifies novel markers and subpopulations in complex tissues

Niya Wang, Eric P. Hoffman, Lulu Chen, Li Chen, Zhen Zhang, Chunyu Liu, Guoqiang Yu, David M. Herrington, Robert Clark, and Yue Wang

**Sample dimension deduction.** Concerning the potential loss of information with dimension reduction (*e.g.*, PCA), the actual loss of information should be theoretically minimal when dealing with multiple samples while the number of underlying true sources is limited to *K*. Consider the generic linear latent variable model **X=AS**, for *K*-source mixed samples, the rank of matrix **S** is *K*; accordingly, since **A** is a full rank matrix, the rank of the resulting matrix **X** remains *K*. Geometrically, the scatter plot of the *K*+ pseudo samples (constructed from PCA dimensional reduction as the linear combinations of the original samples) is intrinsically *K*-dimensional while living in a multidimensional 'original' sample space. When all underlying assumptions are valid there should be no, or minimal, loss of information with eigenvalue decomposition.

It is important to note that sample dimension reduction is conducted before data deconvolution. Provided that the loss of the observed information is minimal, the separability of patterns is not compromised and is ideally handled by the subsequent signal deconvolution process. The extensive simulation studies that investigated the information loss by eigenvalue decomposition in a Gaussian noise environment support this expectation.

**Marker gene concept.** Subpopulation-specific marker (or marker gene) is a widely accepted and adopted concept [1-8]. Subpopulation-specific markers are defined as the markers whose expression values are primarily enriched in a specific subpopulation [1,4,6,9]. Concerning the key assumption on the existence of subpopulation-specific markers for the unsupervised deconvolution approach, the justification is twofold. First, the assumption on the coexistence of distinct subpopulations and subpopulation-specific marker genes is logical, since the definition of a distinct subpopulation would require a set of MGs that are 'unique' to that subpopulation. Hence, a subpopulation may not be considered 'distinct' if it does not have associated unique MGs. Second, since in our newly proved theorems, the coexistence of

distinct subpopulations and subpopulation-specific marker genes is proven to be both a sufficient and a necessary condition for a successful deconvolution. Thus, if the assumption on the coexistence of distinct subpopulations and subpopulation-specific marker genes is not true, a successful deconvolution of mixed expressions (validated by the ground truth) is not possible. If an accurate deconvolution of mixed expressions is successfully achieved, the coexistence of distinct subpopulations and subpopulation-specific marker genes would be guaranteed.

The indices and expression levels of markers are condition dependent. Thus, unsupervised methods for detecting condition-dependent and subpopulation-specific markers are preferred over supervised methods. For example, since expression data are often noisy and the relevant literature is often incomplete, simply using *a priori* markers may not provide accurate information for data deconvolution [3]. Moreover, from several lines of evidence including our own work in Novartis with sorted cells [10], we know that there are other populations that also express the CD14 monocyte marker. Specifically, CD14 is also significantly expressed in CD15 positive cells, and exhibits a moderate expression level in both CD4 positive and CD56 positive cells. Consequently, sorted CD14 positive cells represent a mixture of monocytes and some fraction of cells expressing CD15, CD4, and CD56. Furthermore, CD15, CD4, and CD56 cells also express the CD14 epitope. This is not a technical artifact but inherent to using CD14 as a "specific" marker for monocytes. Kuhn *et al.* [3] state that "*Monocyte-specific genes (e.g. FCN1, CD163) were expressed at very low levels and we did not further consider the contribution of monocytes*." The likely reason for this statement is that the markers used for monocytes are not 'unique' when using a supervised approach. Since monocytes play an important roles in their study, the exclusion of monocytes in the analysis may affect the accuracy of relevant estimation on other components, *e.g.*, the correlation coefficient between the estimated and actually proportions of lymphocytes varies between 0.58 and 0.8.

**Comparative studies on dataset GSE11058.** Data are analyzed using Microarray Suite version 5.0 (MAS 5.0) with Affymetrix default analysis settings. The signal intensity was normalized using global scaling [11], where the trimmed mean target intensity of each array was arbitrarily set to 500. The representative peer method by Zuckerman1 *et al.* [8] is a supervised method [1,2], where the authors explicitly state that "*The algorithm that we propose requires as input purified gene-expression reference signatures for each cell-type*." Directly using the mixed gene expression profiles, CAM blindly detected 301 markers associated with the four pure subpopulations. From the heat map of marker gene expression values given in Fig. 4b,

which shows the pattern of exclusive enrichment in a particular subpopulations, we can see the evidence for the existence of subpopulation-specific markers and for CAM's ability to identify them blindly and correctly.

In contrast, when using a supervised method to analyze heterogeneous tissue, the user is required to have some *a priori* knowledge regarding the nature of the tissue and its possible subpopulation constituents. Usually, purified signatures of the candidate subpopulations are found in public repositories [8], even though they may not be acquired under the same condition as the dataset now being interrogated [3]. In the cell line analysis, the mixed expression profiles were acquired from mixtures of RNAs obtained from 4 cell lines; there was no opportunity for microenvironmental or developmental factors to influence the gene expression of the reference and the constituent populations [6].

The peer method proposed by Guojoux et al. [2] is again a supervised method. Each of the seven NMF algorithms requires and uses *a priori* markers at some stage of the deconvolution process. The authors acknowledged [2] "*the limitation that marker probe sets are selected based on pure samples, while this would not be possible in a real setting where these samples would not be available*." The best result obtained by a semi-supervised NMF method achieves a correlation coefficient (between the estimate and true expression profiles) of 0.91, lower than the correlation coefficient of 0.95 achieved by our unsupervised CAM method on the same dataset.

**CAM analysis on cell cycle dataset.** This analysis uses the CDC28 profile from Cho *et al.* [12], where cdc28-13 cells were collected at 17 time points taken at 10 min intervals, covering nearly two full cell cycles (synchronous yeast cells growing in $G_1$, S, $G_2$, M, and M/$G_1$ phases). There are 6,208 transcripts in total. In identifying cell cycle relevant genes, a comparative study reported by de Lichtenberg *et al.* [13] reveals that Spellman's method [14] outperforms other methods in identifying periodically expressed genes. In Spellman's paper, Fourier transformation was used to assign a CDC score to each gene, and genes with CDC score higher than a threshold were identified as cell cycle regulated genes. The authors required the CDC score threshold to be exceeded by 90% of known cell cycle regulated genes (104 genes verified by traditional method), and accordingly, 800 genes were identified as cell cycle regulated by this method. The 800 genes were identified using three datasets: alpha factor, CDC15 and CDC28. Our experiments use CDC28, because the alpha factor or CDC15 contains too many missing values. In the 800 genes, 113 genes belong to M/G1 phase; 300 genes belong to G1 phase; 121 genes belong to G2 phase; 71 genes belong to S phase; 195 genes belong to M

phase. Among the 800 genes selected by Spellman et al. [14], 781 genes can be found in CDC28.

Among the 187 OVEDEGs blindly detected by CAM, 22 genes belong to the 104 verified cell cycle regulated genes [14,15], and 114 genes are overlapped with 781 genes in CDC28. Specifically, the marker genes detected by CAM include: YDL155W, YDR356W, YER011W, YFR028C, YJL092W, YLR210W, YOR233W, YPR141C, YBR067C, YBR010W, YDR225W, YAR018C, YGL116W, YGL145W, YGR092W, YGR108W, YHR152W, YIL106W, YLR131C, YMR001C, YOR058C, YPR119W, YBR083W, YDL179W, YGR044C, YJL157C, YKL185W, YLR079W, YLR286C, YNL327W, YAR007C, YBL035C, YBR278W, YDL003W, YDL102W, YDL197C, YDR097C, YER070W, YER095W, YGR109C, YJL115W, YJL187C, YKL045W, YKL113C, YLR103C, YML021C; YNL102W, YNL289W, YNL312W, YNR044W, YOR074C, YPL153C, YPL256C, YPR120C, YPR175W.

## References


1 Abbas, A. R., Wolslegel, K., Seshasayee, D., Modrusan, Z. & Clark, H. F. Deconvolution of blood microarray data identifies cellular activation patterns in systemic lupus erythematosus. *PLoS One* **4**, e6098, doi:10.1371/journal.pone.0006098 (2009).

2 Gaujoux, R. & Seoighe, C. Semi-supervised Nonnegative Matrix Factorization for gene expression deconvolution: a case study. *Infect Genet Evol* **12**, 913-921, doi:S1567-1348(11)00293-0 (2012).

3 Kuhn, A. *et al.* Cell population-specific expression analysis of human cerebellum. *BMC Genomics* **13**, 610, doi:10.1186/1471-2164-13-610 (2012).

4 Kuhn, A., Thu, D., Waldvogel, H. J., Faull, R. L. & Luthi-Carter, R. Population-specific expression analysis (PSEA) reveals molecular changes in diseased brain. *Nat Methods* **8**, 945-947, doi:nmeth.1710 (2011).

5 Lu, P., Nakorchevskiy, A. & Marcotte, E. M. Expression deconvolution: a reinterpretation of DNA microarray data reveals dynamic changes in cell populations. *Proc Natl Acad Sci U S A* **100**, 10370-10375, doi:10.1073/pnas.1832361100 (2003).

6 Qiao, W. *et al.* PERT: a method for expression deconvolution of human blood samples from varied microenvironmental and developmental conditions. *PLoS Comput Biol* **8**, e1002838, doi:10.1371/journal.pcbi.1002838 (2012).

7 Stuart, R. O. *et al.* In silico dissection of cell-type-associated patterns of gene expression in prostate cancer. *Proc. Natl. Acad. Sci.* **101**, 615-620 (2004).

8 Zuckerman, N. S., Noam, Y., Goldsmith, A. J. & Lee, P. P. A self-directed method for cell-type identification and separation of gene expression microarrays. *PLoS Comput Biol* **9**, e1003189, doi:10.1371/journal.pcbi.1003189 (2013).

9 Yu, G. *et al.* PUGSVM: a caBIG analytical tool for multiclass gene selection and predictive classification. *Bioinformatics* **27**, 736-738, doi:10.1093/bioinformatics/btq721 (2011).

10 Gong, T. *et al.* Optimal deconvolution of transcriptional profiling data using quadratic programming with application to complex clinical blood samples. *PLoS One* **6**, e27156, doi:10.1371/journal.pone.0027156 (2011).

11 Wang, Y., Lu, J., Lee, R., Gu, Z. & Clarke, R. Iterative normalization of cDNA microarray data. *IEEE Trans Inf Technol Biomed* **6**, 29-37 (2002).

12 Cho, R. J. *et al.* A genome-wide transcriptional analysis of the mitotic cell cycle. *Mol Cell* **2**, 65-73 (1998).

13 de Lichtenberg, U. *et al.* Comparison of computational methods for the identification of cell cycle-regulated genes. *Bioinformatics* **21**, 1164-1171, doi:10.1093/bioinformatics/bti093 (2005).

14 Spellman, P. T. *et al.* Comprehensive identification of cell cycle-regulated genes of the yeast Saccharomyces cerevisiae by microarray hybridization. *Mol Biol Cell* **9**, 3273-3297 (1998).

15 Johansson, D., Lindgren, P. & Berglund, A. A multivariate approach applied to microarray data for identification of genes with cell cycle-coupled transcription. *Bioinformatics* **19**, 467-473 (2003).
